# Identifying Alternative Hyper-Splicing Signatures in MG-Thymoma by Exon Arrays

**DOI:** 10.1371/journal.pone.0002392

**Published:** 2008-06-11

**Authors:** Lilach Soreq, Adi Gilboa-Geffen, Sonia Berrih-Aknin, Paul Lacoste, Ariel Darvasi, Eyal Soreq, Hagai Bergman, Hermona Soreq

**Affiliations:** 1 Department of Physiology, The Hebrew University, Hadassah Medical School, Jerusalem, Israel; 2 Department of Biological Chemistry, The Faculty of Science, The Hebrew University of Jerusalem, Jerusalem, Israel; 3 University Paris-Sud, UMR 8162, Hôpital Marie Lannelongue, Le Plessis Robinson, Paris, France; 4 CNRS, Le Plessis Robinson, Paris, France; 5 Department of Genetics, The Faculty of Science, The Hebrew University of Jerusalem, Jerusalem, Israel; 6 Bezalel Academy of Arts and Design, Jerusalem, Israel; Lehigh University, United States of America

## Abstract

**Background:**

The vast majority of human genes (>70%) are alternatively spliced. Although alternative pre-mRNA processing is modified in multiple tumors, alternative hyper-splicing signatures specific to particular tumor types are still lacking. Here, we report the use of Affymetrix Human Exon Arrays to spot hyper-splicing events characteristic of myasthenia gravis (MG)-thymoma, thymic tumors which develop in patients with MG and discriminate them from colon cancer changes.

**Methodology/Principal Findings:**

*W*e combined GO term to parent threshold-based and threshold-independent *ad-hoc* functional statistics with in-depth analysis of key modified transcripts to highlight various exon-specific changes. These denote alternative splicing in MG-thymoma tumors compared to healthy human thymus and to in-house and Affymetrix datasets from colon cancer and healthy tissues. By using both global and specific, term-to-parent Gene Ontology (GO) statistical comparisons, our functional integrative *ad-hoc* method allowed the detection of disease-relevant splicing events.

**Conclusions/Significance:**

Hyper-spliced transcripts spanned several categories, including the tumorogenic ERBB4 tyrosine kinase receptor and the connective tissue growth factor CTGF, as well as the immune function-related histocompatability gene HLA-DRB1 and interleukin (IL)19, two muscle-specific collagens and one myosin heavy chain gene; intriguingly, a putative new exon was discovered in the MG-involved acetylcholinesterase *ACHE* gene. Corresponding changes in spliceosome composition were indicated by co-decreases in the splicing factors ASF/SF_2_ and SC35. Parallel tumor-associated changes occurred in colon cancer as well, but the majority of the apparent hyper-splicing events were particular to MG-thymoma and could be validated by Fluorescent In-Situ Hybridization (FISH), Reverse Transcription–Polymerase Chain Reaction (RT-PCR) and mass spectrometry (MS) followed by peptide sequencing. Our findings demonstrate a particular alternative hyper-splicing signature for transcripts over-expressed in MG-thymoma, supporting the hypothesis that alternative hyper-splicing contributes to shaping the biological functions of these and other specialized tumors and opening new venues for the development of diagnosis and treatment approaches.

## Introduction

Changes in gene expression, and particularly in alternative splicing patterns are often disease-associated [1], and aberrant alternative splicing (hyper-splicing) is one of the characteristics of cancer cells [2], as well as of inflammation and autoimmune muscle diseases[3]. The vast majority of human genes (>70%) are alternatively spliced [4], [5], and 75% of alternative splicing events affect coding regions, yielding subtle amino acid substitutions, removal of protein motifs or protein truncations [6]. This can alter protein structures, yield cell-specific protein patterns [7] and enlarge protein versatility in a tissue-specific manner [6]. However, tumor specific signatures of alternative hyper-splicing are still lacking, primarily since alternative splicing studies are based on expressed sequence tags (EST) or mRNA sequences [8], [9]. Poor coverage of low abundance transcripts [4], and uncovered tissues, disease states and developmental stages [10] hence call for the development of methodologies for identifying alternative hyper-splicing signatures in specific tumor types.

In search for tumor-specific alternative hyper-splicing signatures, we selected MG-thymoma, an epithelial tumor of the thymus gland [11] where lymphoid precursor cells differentiate into mature T-lymphocytes [12]. About one in three of all thymoma patients develop Myasthenia Gravis (MG), a neuromuscular autoimmune disease characterized by abnormal neuromuscular transmission [13], [14]. In MG, auto-immune antibodies against the muscle nicotinic acetylcholine receptor are accompanied by thymocytes hyperplasia. MG involves loss of acetylcholine receptors that initiate muscle contraction, which results in progressive muscle weakness. Overall, more than 60% of MG patients present a pathological thymus, including thymic hyperplasia in about 50% of patients and thymoma in 10 to 20% [14]. Understanding the role of alternative hyper-splicing of tumor-related, muscle-specific and immune function genes in the etiology of MG-thymoma can provide better diagnosis for patients and offer more hope for their treatments [15].

Linkage between regulated genes and the corresponding transcript quantities highlights tumor over-expressed transcripts as preferred targets for identifying hyper-splicing signatures [16]. We have recently used UniGene microarrays [17] to identify such over-expressed transcripts. However, those microarrays primarily detect the 3′-end of studied transcripts, disregarding transcript levels. Thus, Agilent [18] 60-mer probes are located between 150 to 1100 bps from the target transcript 3′-ends [18], whereas the 11 25-mer probes per gene of Affymetrix GC U133 Plus Arrays are located in a 600 bp range from these genes 3′-end [19]. Some of these limitations were overcome in the high-density lithography-based Affymetrix exon arrays [10], [20], where each transcript is interrogated by a large number of 25-mer probes [10] (50 in average), located according to its complete RefSeq annotation, and amplified RNA is prepared to cover the entire length of the analyzed transcripts. The massive increase in probes (∼5.6 million), along with exon length-dependent probe numbers, enable unprecedented resolution into these genomic units. This ensures more robust detection of gene-level transcription changes [10] and allows the discovery of potentially new transcripts and novel, predicted exons.

At the functional level, alternative hyper-splicing can modify tumor properties, since gene products may play roles in multiple, often seemingly unrelated, routes. To approach these processes, tools for functional analyses of microarray data have been developed [21]. These primarily involved *post-hoc* functional analyses such as T-test, ANOVA [22] or clustering analyses [23] (i.e first finding lists of changed genes, by various computational methods, and then conducting functional enrichment analysis on these lists, e.g. EASE [24] and MAPPFinder [25]). However, translating a list of differentially expressed genes using annotation databases suffers from several limitations. Primarily, this approach overlooks transcripts that are involved in several biological processes. In addition, most downstream analysis tools for gene lists enable analyses of only one ontology abstraction level [21]. To address these difficulties, methods that allow *ad-hoc* detection of changed functional GO categories have been evolving. These largely use threshold-based statistical approaches (i.e. binomial, hypergeometric, chi-square or Fisher's exact test) that detect relative enrichments of gene categories given a population of genes [21]. However, they involve high incidence of false negatives that result from a unified, often arbitrary threshold [26], [27].

To increase the range of solutions and enable detection of subtle and non-trivial changes, several threshold-free methods were developed (for example, GSEA [28] and a program based on semantic similarities among GO terms [29]). These are based on the Kolmogorov-Smirnov (KS) goodness-of-fit test [27], [28], [30], [31], which prevents neglecting most of the collected data but may be more error-prone to false positives. Although rank-based methods have been suggested [32], [33], the important conceptual advantage of threshold-freedom lies in considering gene expression data simultaneously, without the uncertainty associated with prior gene list extraction [26]. Combining both discrete and continuous data analyses to detect changed functional gene GO categories from expression data can thus provide several advantages for analyzing microarrays [27].

We applied an *ad-hoc* method for multileveled testing of GO categories enrichment in exon array-derived data sets by combining a KS and Fisher Exact (Hypergeometric), with detailed term-to-parent statistical analysis on the GO categories represented on the arrays. First, we analyzed our in-house MG-thymoma study and the Affymetrix colon cancer [10] exon microarray data set, which identified tumor-specific alternative splicing events relevant to both tumors or specific to one of them. In-depth analyses of exon-specific alterations involved several key transcripts from the tumor-related, immune function and muscle-characteristic GO categories and the MG-related *ACHE* gene, which is ubiquitously expressed, undergoes alternative splicing at both termini (3′ and 5′) and contributes to many different biological processes [34].

To study the contribution of alternative splicing to tumor biology, we developed a novel approach for identifying alternative hyper-splicing signatures. Using human exon microarrays, we compared MG-thymoma tumors from patients with MG to Affymetrix datasets from healthy tissues and colon cancer. Combined ad-hoc and *post-hoc* statistics with in-depth analysis of key transcripts and FISH, RT-PCR and mass spectrometry followed by peptide sequencing validation revealed pronounced alternative hyper-splicing in several gene categories modified in MG-thymoma tumors. These spanned tumorogenic, immune function and muscle-specific transcripts, involved reduced expression of the splicing factors ASF/SF_2_ and SC35 and extended into the discovery of a putative new exon in the MG-involved *ACHE* gene. Together, these findings support the notion of a major contribution of alternative hyper-splicing to MG-thymoma features, opening new venues for diagnosis and treatment of specific tumor types, and revealed specific tumor-type alternative splicing signatures reflecting MG-thymoma and colon cancer properties.

## Results

**Figure 1:**
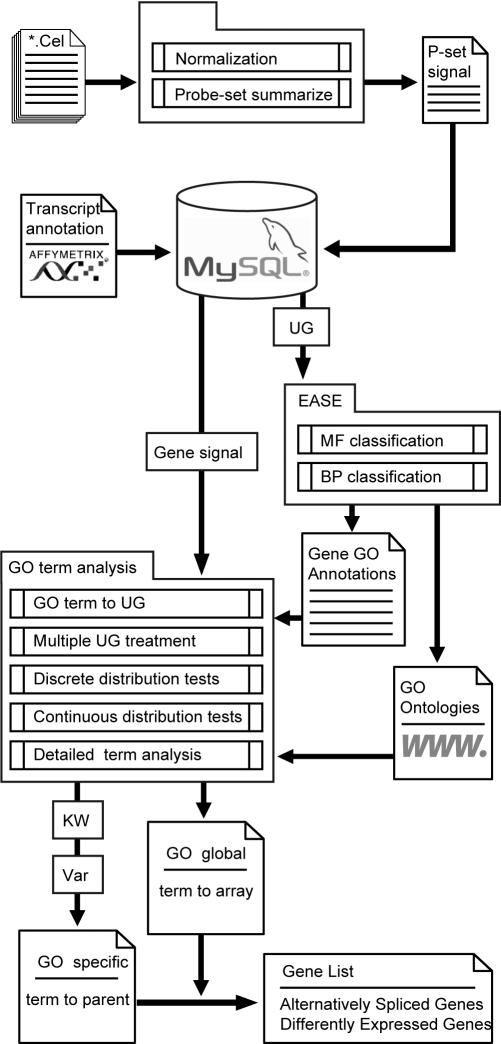
Exon array analysis workflow-gene-level signals are calculated from normalized exon array CEL files by summarizing probe sets signals (either by PLIER or any other gene level summary method). MySQL database stores exon array annotation files and expression data at both the exon (probe-set) and gene levels. Gene-level data, UniGene codes, as well as GO (www.geneontology.org) ontology files and EASE [24] serve to perform functional analysis. The functional analysis detects both globally changed GO BP and MF terms using continuous (KS) and discrete (Fisher exact) tests, as well as specific changes of terms compared to their parent terms. The specific functional analysis results are used to target specific genes that exhibited alternative splicing events or general differential expression by presenting fold change on core probe sets of detected categories.

To identify tumor-specific changes in gene expression, we used in-house data of cortical thymoma tumors from MG patients and normal thymuses and the Affymetrix sample data set [20] of colon cancer versus normal colon and other healthy tissues. We applied an integrative functional *ad-hoc* analysis method to gene-level summary signals (median core probe sets or PLIER signal estimates [10], [20]). Each term was compared to the global array population and to all its direct parent GO terms as well as to the root of the tree: Molecular Function (MF) or Biological Process (BP) (Figure 1). Additional statistical tests of location involved the Kruskal-Wallis and variance tests. Within the categories that changed specifically compared to their parent GO terms we searched for genes containing core probe sets that changed by more than 4-fold between MG-thymoma to control thymuses.

Iter-PLIER gene-level data of Affymetrix colon cancer data set [20] (data will be given upon request) served for comparison of the detected genes. In addition, we tested the sensitivity and specificity of our *ad-hoc* approach by conducting it on the colon cancer data set (results under Table S1) and found a wide overlap of tumor to normal tissue data compared to other colon cancer 3′ microarray analyses and one proteomic data set. This revealed a higher level of accuracy, superior to others' published data sets in both *post-* and *ad*-*hoc* approaches [20], [24], [35]–[37] (Text S1). We detected 100% of the categories identified by all the other methods, at the significance levels of 0.05 and 0.001. Our total findings overlapped with others ranged at 31–90%, with the closest (90.91%) achieved by comparison to *post-hoc* analysis we applied on the Affymetrix colon cancer exon array data set [20], [24], [35]–[37], making false negatives unlikely. Importantly, 56.63% of the categories detected by us at significance level of 0.01 were also detected by one or more of the compared methods (Text S1), which exhibited lower overlap ratios among them (26.74–43.97%). One functional bioinformatics *post-hoc* method was similar to ours in detection ratios compared to the other methods (56.76%) [35].

### Global continuous and discrete term analyses reveal MG-thymoma affected GO terms

To search for the molecular changes that underlie MG-thymoma we applied our *ad-hoc* functional analysis and compared each GO term to the global array population. We observed ample changes in both BP and MF categories (Figure S1). Noticeably, more categories decreased according to both approaches; compatible with the exon and gene level changes, also when compared to the colon cancer data set (Text S2, Figure S2) and with the decrease in GO categories in hyperplasic thymuses [38]. Permutations of both MG-thymoma and colon cancer data sets verified those global expression change difference (Figure S3). In both MG-thymoma and colon cancer data sets, we found differences between expression changes in the 3′ compared to 5′ at the whole genome level (Text S2, Figure S4–5). Noticeably, MG-thymomas showed increase in *muscle contraction* (P<0.05 by both discrete and continuous methods, Table S2) both in this and in our previous study [38]. Totally, 85 BP and 97 MF categories exhibited a change in MG-thymomas compared to healthy thymuses. Predictably, more categories were detected by the continuous approach as compared with discrete analyses (Text S2). Nevertheless, decreased MF categories showed an almost total overlap between the two methods.

### Specific hierarchical term to parent analyses in MG-thymoma

Each term represented by the array transcripts was compared to its direct parent terms in the GO tree, as well as to its tree root (MF/BP), using both continuous and discrete data analyses, as well as tests for location and dispersion (Table S3). Several key categories were found to be changed. These included *RNA binding*, which changed (Figure S6) compared to two parent terms (MF): *transcriptional repressor activity* (decreased, P = 0.01 in the continuous method, with changed dispersion and location, P<0.05) and compared to *complement receptor activity* (with a change in dispersion, P<0.01). *Cell cycle* terms showed both decreases and increases compared to its direct parent, *cellular physiological process* (P<0.005, BP). More GO categories changed compared to their direct parent terms (i.e specific analysis) than compared to the whole array population (i.e global analysis), identifying specific GO levels that changed and pointing at specific disease-related genes.

### Finding disease-relevant genes in MG-thymoma by combining term to parent analysis with threshold on core exons

**Figure 2:**
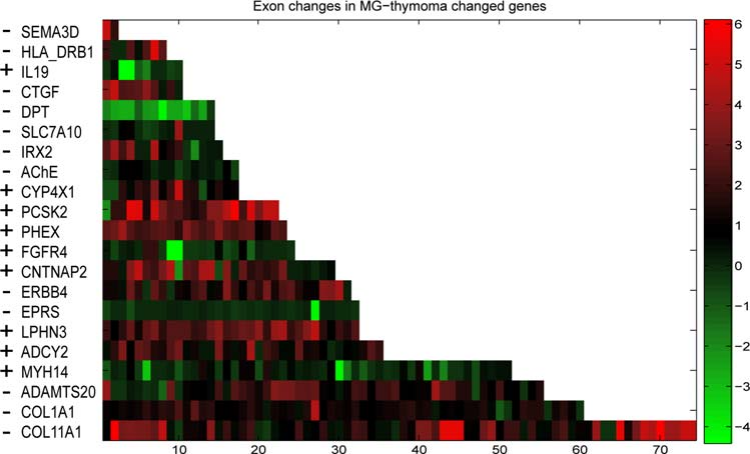
MG-thymoma targeted genes identified as subject to major changes in MG-thymoma tumors by specific, term-to-parent, ad-hoc combined statistical analyses and ACHE. The color scale (right) presents fold changes (log base 2), and each core exonic probe set is presented as a single band. The strand of each gene is denoted (as+for forward or-for reverse). The exons are ordered from 5′ to 3′ for genes on the+strand and from 3′ to 5′ for genes on the-strand, such as ACHE. Note that most transcripts show mixed patterns of changes (both increases and decreases), reflecting alternative splicing.

**Figure 3:**
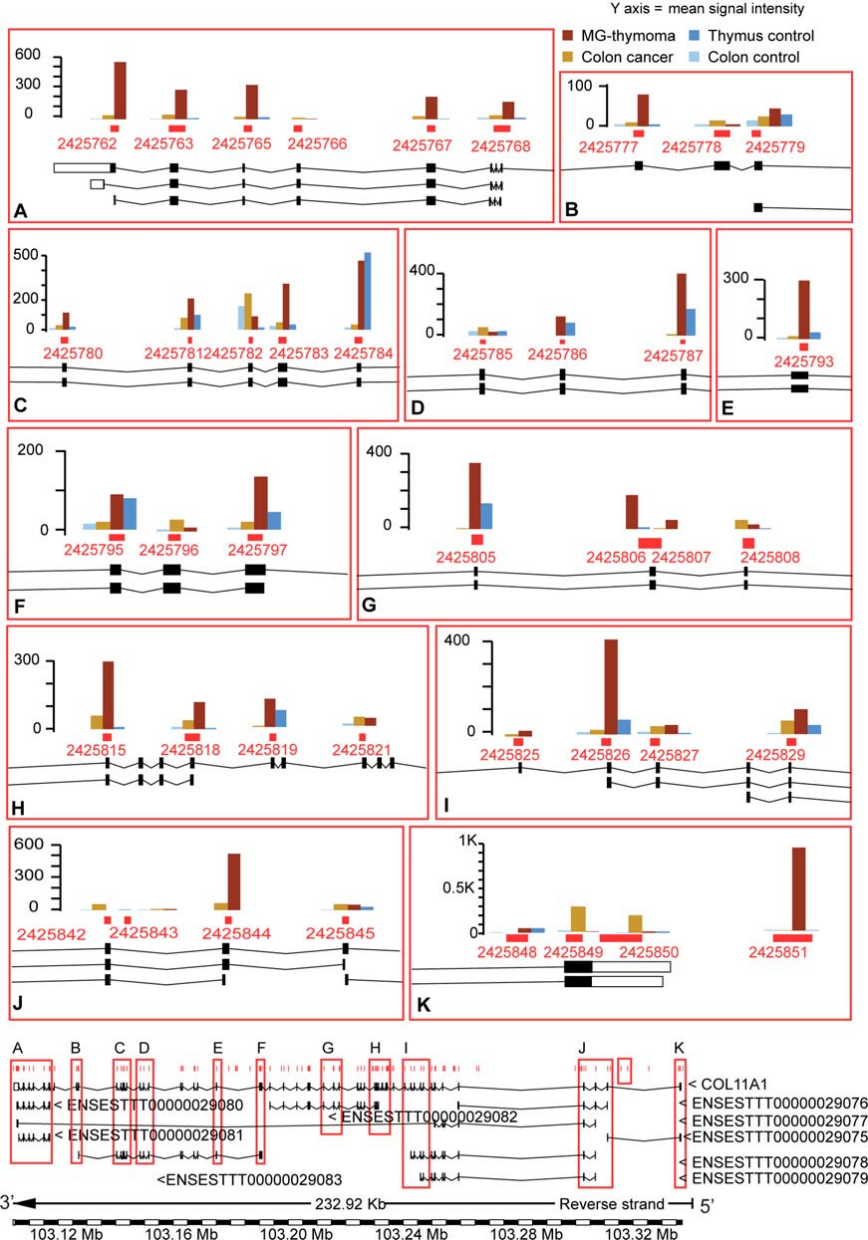
Detailed expression map of COL11A1 in MG-thymoma and colon cancer. The known transcript variant of COL11A1 (adopted from ENSEMBL [7]) is shown. Additional ENSEMBL EST-based transcripts [6] of COL11A1 are shown below it. The gene was up-regulated in MG-thymoma compared to both healthy thymus, colon cancer and healthy colon (A-J). An evidence for alternative start site in the thymus is apparent (K).

**Figure 4:**
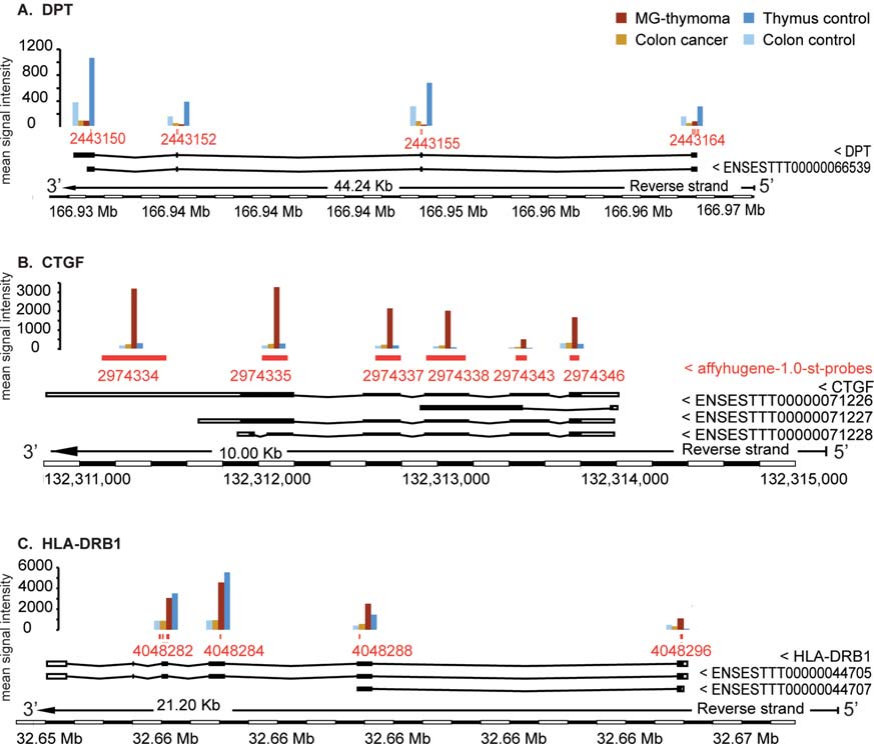
Detailed expression map of HLA-DRB1, CTGF and DPT in MG-thymoma and colon cancer. (A) DPT is down regulated in both MG-thymoma and colon cancer compared to healthy thymus and colon samples. (B) CTGF is up-regulated only in MG-thymoma. (C) HLA-DRB1 is up-regulated in the thymus compared to colon tissues. It exhibits the existence of at least two transcript variants predicted by ENSEMBL [6], one that includes exons 1 and 2 and the other, two additional exons.

We used the results of our specific term analysis to search for core exons with a cutoff threshold of 4–6 fold difference between patients to healthy subjects. We identified 20 genes belonging to the tumor-related, immune function and muscle-specific categories (Table 1). To these we added *ACHE,* which expresses at low levels but is highly relevant to MG [38], [39]. Figure 2 presents these changes as color-coded clusters highlighting the alternative splicing modifications. The MF and BP categories to which the genes belong and corresponding term-to-parent changes are listed under Table 2 and 3. *COL11A1* (Figure 3), *extracellular matrix structural constituent,* changed compared to its two direct parents (Figure S6). *COL11A1* was up regulated in MG-thymoma (4–6 fold in a large number of core probe-sets) and more moderately in colon cancer compared to healthy colon (Figure 3A–J J and Text S3). Its first known exon was expressed in the colon but not the thymus, whereas in MG-thymoma an extended probe set located just before the first exon was highly expressed (Figure 3K), indicating alternative splicing in this large (0.2 Mb) gene. Another collagen, *COL1A1* (*skeletal development,* BP), is notably involved in chronic inflammation [40]. *COL1A1* was up-regulated in MG-thymoma compared to healthy thymuses and in colon cancer compared to healthy colons. The tumor-related genes included the tyrosine kinase receptor *ERBB4* (*cell proliferation*, *nucleotide binding*, MF) which was up-regulated in MG-thymoma (Text S3) and is de-regulated in many human tumors [41]. In contrast, the growth factor dermatopontin (*DPT*), which belongs to *cell adhesion* and *protein binding*, was down-regulated in both cancer types (Figure 4A).

**Table 1:** Targeted MG-thymoma genes

Gene name	Ensamble ID	Transcript cluster ID	Full Gene name
*ADAMTS20*	ENSG00000173157	3451558	ADAM metallopeptidase with thrombospondin type 1 motif, 20
*ADCY2*	ENSG00000078295	2800711	Adenylate cyclase 2
*CNTNAP2*	ENSG00000174469	3029900	Contactin associated protein-like 2
*COL1A1*	ENSG00000108821	3762198	Collagen, type I, alpha 1
*COL11A1*	ENSG00000060718	2425756	Collagen alpha-1(XI) chain precursor
*CTGF*	ENSG00000118523	2974330	Connective tissue growth factor
*CYP4X1*	ENSG00000186377	2334986	Cytochrome P450, family 4, subfamily X, polypeptide 1
*DPT*	ENSG00000143196	2443120	Dermatopontin
*EPRS*	ENSG00000136628	2456746	Glutamyl-prolyl-tRNA synthetase
*ERBB4*	ENSG00000178568	2597552	V-erb-a erythroblastic leukemia viral oncogene homolog 4 (avian)
*FGFR4*	ENSG00000160867	2842911	Fibroblast growth factor receptor 4
*HLA-DRB1*	ENSG00000196126	4048265	Major histocompatibility complex, class II, DR beta 1
*IL19*	ENSG00000142224	2376988	Interleukin 19
*IRX2*	ENSG00000170561	2846522	Iroquois homeobox protein 2
*LPHN3*	ENSG00000150471	2728938	Latrophilin 3
*MYH14*	ENSG00000105357	3839206	Myosin, heavy polypeptide 14
*PCSK2*	ENSG00000125851	3877892	Proprotein convertase subtilisin/kexin type 2
*PHEX*	ENSG00000102174	3971451	Phosphate regulating endopeptidase homolog, X-linked (hypophosphatemia, vitamin D resistant rickets)
*SEMA3D*	ENSG00000153993	3059667	Sema domain, immunoglobulin domain (Ig), short basic domain, secreted, (semaphorin) 3D
*SLC7A10*	ENSG00000130876	3858907	Solute carrier family 7, (neutral amino acid transporter, y+ system) member 10
*ACHE*	ENSG00000087085	3064361	Acetylcholinesterase

Detailed core probe sets expression graphs are under S12.

**Table 2:** MF Specific changed GO terms compared to their parent GO terms in MG-thymoma

Molecular Function (GO ID) (N)	Parent (GO ID) (N)	C	D	KW	VAR
Transcription corepressor activity (3714) (16)	Transcription cofactor activity (3712) (30)	↑		**√**	
RNA binding (3723) (83)	Transcriptional repressor activity (16564) (101)	↓		**√**	**√**
Motor activity (3774) (17)	Complement receptor activity (4875) (19)	↓		**√**	
Defense/immunity protein activity (3793) (9)	Complement receptor activity (4875) (11)	↓		**√**	**√**
Cytokine activity (5125) (33)	Protein binding (5515) (378)	↑	↑	**√**	
Ion channel activity (5216) (39)	Alpha-type channel activity (15268) (48)	↓		**√**	
Apoptosis regulator activity (16329) (29)	Molecular function	↑			
Receptor activity (4872) (179)	Signal transducer activity (4871) (377)		↑		
Ligase activity (16874) (68)	Catalytic activity (3824) (615)		↑		
Transcriptional repressor activity (16564) (18)	Transcription regulator activity (30528) (147)	↑	↑	**√**	
Extracellular matrix structural constituent ( 5201) (27)	Structural molecule activity (5198) (79)				**√**
Extracellular matrix structural constituent ( 5201) (27)	Growth factor activity (8083) (47)				**√**
Nucleotide binding ( 166) (199)	Binding (5488) (968)	↓	↓		**√**
Magnesium ion binding (287) (21)	Molecular function	↑			
Magnesium ion binding (287) (21)	Metal ion binding (46872) (177)	↑	↓		
Transcription factor activity (3700) (78)	Transcription regulator activity (30528) (135)		↑		**√**
Transcription factor activity (3700) (78)	Complement receptor activity (4875) (80)				**√**
Protein binding (5515) (378)	Binding (5488) (968)		↑↓		
Protein binding (5515) (378)	Metal ion binding (46872) (508)		↓		
DNA replication (6260) (15)	DNA metabolism (6259) (50)				**√**
Binding (5488) (968)	Molecular function	↓			
ATP binding (5524) (176)	Metal ion binding (46872) (348)		↑↓		
monooxygenase activity (4497) (21)	oxidoreductase activity (16491 ) (109)	↑	↑		
Protein serine/threonine kinase activity (4674) (52)	Protein kinase activity (4672) (68)		↓		

Full results of term-to parent analysis are under Table S3.

**Table 3:** BP Specific changed GO terms compared to their parent GO terms in MG-thymoma

Biological Process (GO ID) (N)	Parent (GO ID) (N)	C	D	KW	VAR
M phase of mitotic cell cycle (87) (12 )	M phase (279) (15)			**√**	
Mitotic cell cycle (278) (13 )	Carboxylic acid metabolism (19752) (19)			**√**	
Muscle contraction (6936) (22 )	Taxis (42330) (29)	↑	↓	**√**	
Inflammatory response (6954) (27 )	Response to wounding (9611) (45)	↓		**√**	**√**
Inflammatory response (6954) (27 )	Response to pest\, pathogen or parasite (9613) (36)	↓		**√**	
Immune response (6955) (184 )	Response to pest\, pathogen or parasite (9613) (36)	↓		**√**	
Humoral immune response (6959) (28)	Response to pest\, pathogen or parasite (9613) (36)	↓		**√**	
Cellular defense response (6968) (7 )	Response to pest\, pathogen or parasite (9613) (36)	↓		**√**	
Response to DNA damage stimulus (6974) (10)	Response to pest\, pathogen or parasite (9613) (36)	↓		**√**	
Cell adhesion (7155) (80 )	Biological process	↓		**√**	
tRNA metabolism (6399) (40 )	RNA metabolism (16070) (63)	↓	↑	**√**	**√**
Response to wounding (9611) (20 )	Response to external stimulus (9605) (141)		↓		
Glutamyl-tRNA aminoacylation (6424) (4)	tRNA aminoacylation for protein translation (6418 ) (37)				**√**
Protein complex assembly (6461) (23)	Biological process	↑		**√**	
Morphogenesis (9653) (35)	Development (7275 ) (199)		↑		
Proteolysis (6508) (63)	Biological process	↑			
Skeletal development (1501) (15)	Organ development (48513) (36)	↑			**√**
Cell proliferation (8283) (43)	Cellular physiological process (50875) (380)		↑↓		**√**
Cell proliferation (8283) (43)	Regulation of signal transduction (9966) (52)	↑	↑		
Cell motility (6928) (18)	Biological proces	↓			
Cell motility (6928) (18)	Cellular physiological process (50875) (369)		↓		
Epidermis development (8544) (14)	Biological process	↑		**√**	
Cell differentiation (30154) (20)	Development (7275) (199)	↑			
Cell differentiation (30154) (20)	Cellular process (9987) (1163)	↑			
Electron transport (6118) (67)	Transport (6810) (317)		↑		
Transmission of nerve impulse (19226) (23)	Transport (6810) (317)		↑		

Full results of term-to parent analysis are under Table S3.

Connective tissue growth factor (*CTGF*) was up-regulated in MG-thymoma compared to control thymus, control colon and colon cancer samples (Figure 4B). The low expression levels of its second exon in MG-thymomas may indicate exon skipping. CTGF belongs to 4 modified MF GO categories: *protein binding*, *DNA replication*, *cell motility* and *epidermis development*. Also in *protein binding* as well as in *cell adhesion* and *transmission of nerve impulse* (decreased, BP) is *CNTNAP2*, which exhibited over 4-fold increase in 4 core probe-sets. CNTNAP2 was highly up-regulated in MG-thymoma, exhibited low expression in colon compared to thymus, and was down–regulated in colon cancer (Text S3). Its 3′ exons are expressed only in MG-thymoma and not in healthy thymus, suggesting an alternative terminus. *LPHN3* (also known as *LEC3*), encodes a *receptor activity* member of the latrophilin G-protein coupled receptors (GPCR). *LEC3* was up-regulated in MG-thymoma, but down-regulated in colon cancer (Text S3), Similar to Adenylate cyclase *(ADCY2*, *magnesium ion binding*), and the iroquise homeobox gene *IRX2,* (*transcription factor activity*). In *proteolysis*, we identified the subtilisin *PCSK2*, with 4-6 fold increases in 8 of its core probe-sets in MG-thymoma and increases in colon cancer (Text S3), and the endopeptidases *PHEX* and *ADAMTS20* (*proteolysis*), both up-regulated in MG-thymoma but not in colon cancer (Text S3). In the BP category *immune response* we found the histocompatibility MHC-II complex gene *HLA-DRB1* (Figure 4C). Its expression is significantly higher in thymus compared to colon, with a putative 5′ end-modified HLA-DRB1-002 transcript in MG-thymoma [42]. Interleukin (*IL*) *19* expressed higher in thymus than colon (Text S3) and increased in MG-thymoma. Also, *SEMA3D* (*cell differentiation*) was highly up regulated in MG-thymoma but not colon cancer (Text S3). The solute carrier *SLC7A10* (changed *transport,* BP), an inflammatory and apoptosis modulator [43], increased both in MG-thymoma and in colon cancer, with higher thymus than colon expression (Text S3).

The tRNA synthetase, *EPRS*, expressed higher in thymus than in colon, belongs to 5 specifically changed MF GO categories: *binding*, *ATP binding*, *ligase activity*, *glutamyl-tRNA aminoacylation* and *protein complex assembly*. It was up-regulated in both MG-thymoma and colon cancer, as well as in different cancer types [44]. Also in *ATP binding*, the fibroblast growth factor receptor *FGFR4* showed over 4-fold decrease in two core probe-sets in MG-thymoma (Text S3). *FGFR4* also belongs to *protein serine/threonine kinase activity* and *receptor activity*. The cytochrome P450 *CYP4X1* gene (*monooxygenase activity, electron transport*) increased in MG-thymoma compared to healthy thymuses, with higher thymus than colon expression and increases in colon cancer as well (Text S3).

**Figure 5:**
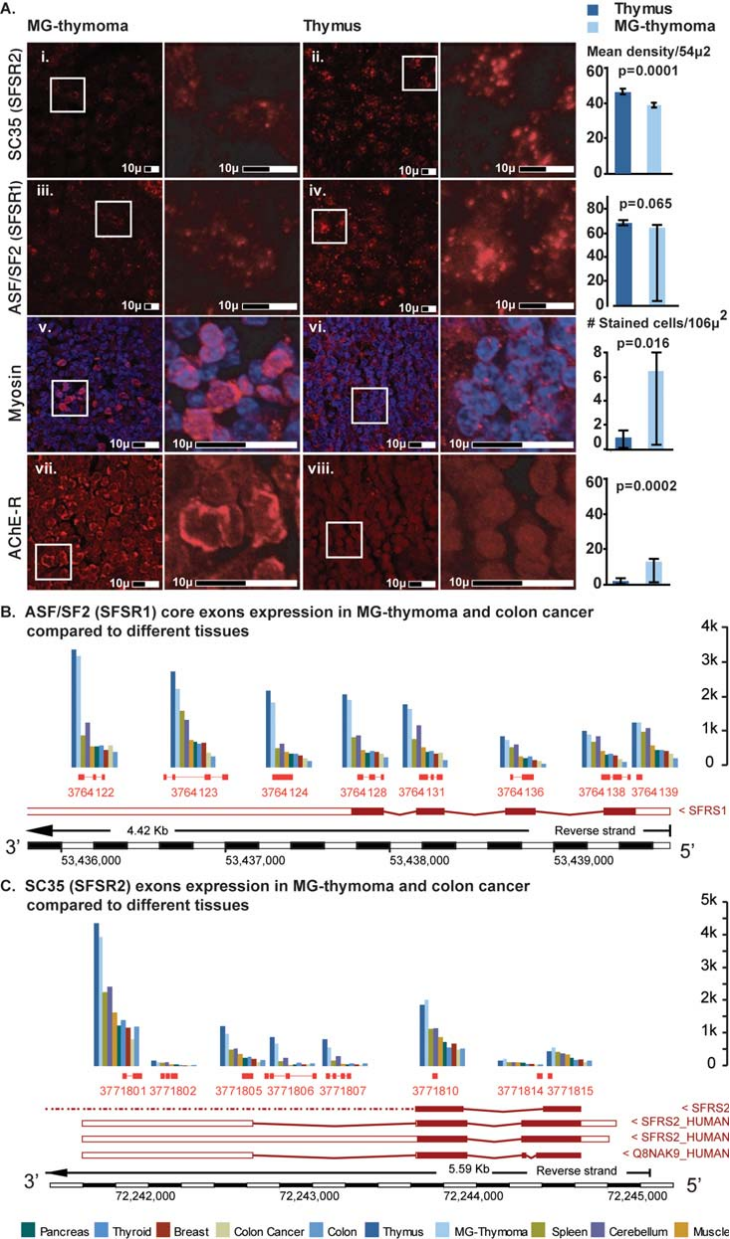
mRNA and protein expression of splicing factors ASF/SF2 and SC35, Myosin and ACHE. Immunohistochemistry indicated (A) Decrease in the mean density of the splicing factors: SR protein ASF/SF2 between MG-thymoma (i) to healthy thymus (ii) (p = 0.065) and SC35 between MG-thymoma (iii) to healthy thymus (iv) (p = 0.0001). The number of myosin expressing cells increased (p = 0.016) in MG-thymoma (v) compared to healthy thymuses (vi). (C) FISH for AChE-R variant indicated a significant increase in the number of stained cells in the MG-thymomic section (vii) compared to healthy thymus (viii) (p = 0.0002). (B) Exonic expression maps of ASF/SF2. All the core exons are shown. MG-thymoma (light blue) exhibited a decrease compared to healthy thymus (dark blue) in both ASF/SF2 (B) and SC35 (C). The expression level in both healthy and cancer thymus were higher then in other tested tissues for both (B and C). The known transcript variant of ASF/SF2 is expressed (C). SC35 exhibits expression of two transcript variants in MG-thymoma compared to thymus samples: one containing exons 1 and 2 alone, and one containing an additional 3′ exon.

The muscle-specific myosin *MYH14* (*nucleotide binding*, *motor activity,* MF) exhibited higher expression levels in the thymus than other examined tissues (Text S3). It exhibited alternative splicing in MG-thymoma tumors compared to healthy thymuses but not between colon cancers to normal colons (Text S3). The initial exons showed higher levels in MG-thymoma where its middle-end exons were higher in normal thymus. This suggests the existence of alternative variants of *MHY14* in MG-thymoma compared to normal thymuses. In addition, we observed a prominent median gene-level increase in MG-thymoma compared to normal thymuses (Text S3). We also detected an increase of Myosin in MG-thymoma using Immunohistochemistry validation with antibody specific to *Myosin* (Figure 5A). Indeed, MG patients produce antibodies against structural muscle proteins, among them myosin [45] and *Myosin* previously showed a change immuno-histochemistry [46].

### Decreased *SC35* and *ASF/SF2* in MG-thymoma

Alternative splicing is modulated by the serine-arginine (SR)-rich protein *SC35* [47], [48] (also known as *SFRS2*, splicing factor, arginine/serine-rich 2, ENSG00000161547), which interacts with different proteins, including *ASF/SF2* (*SFSR1,* ENSG00000136450) [49]. FISH and Immunohistochemistry exhibited *SC35* and *ASF/SF2* decreases in the MG-thymoma tissues (Figure 5A and Figure S7), compatible with the findings of others [50]. Intriguingly, at the exon expression level *ASF/SF2* is higher in both healthy thymus and MG-thymoma, than in all other examined tissues [51]. *SC35* is also higher in thymus than in all other examined tissues. However, both *SC35* and *ASF/SF2* are down-regulated in inflamed muscle [52], and both showed down-regulation in MG-thymoma (Figures 5B, C and Text S3). Moreover, two *SC35* exons show higher expression than the 3′ UTR domain, suggesting alternative splicing.

### A new putative ubiquitously expressed exon in the *ACHE* gene

**Figure 6:**
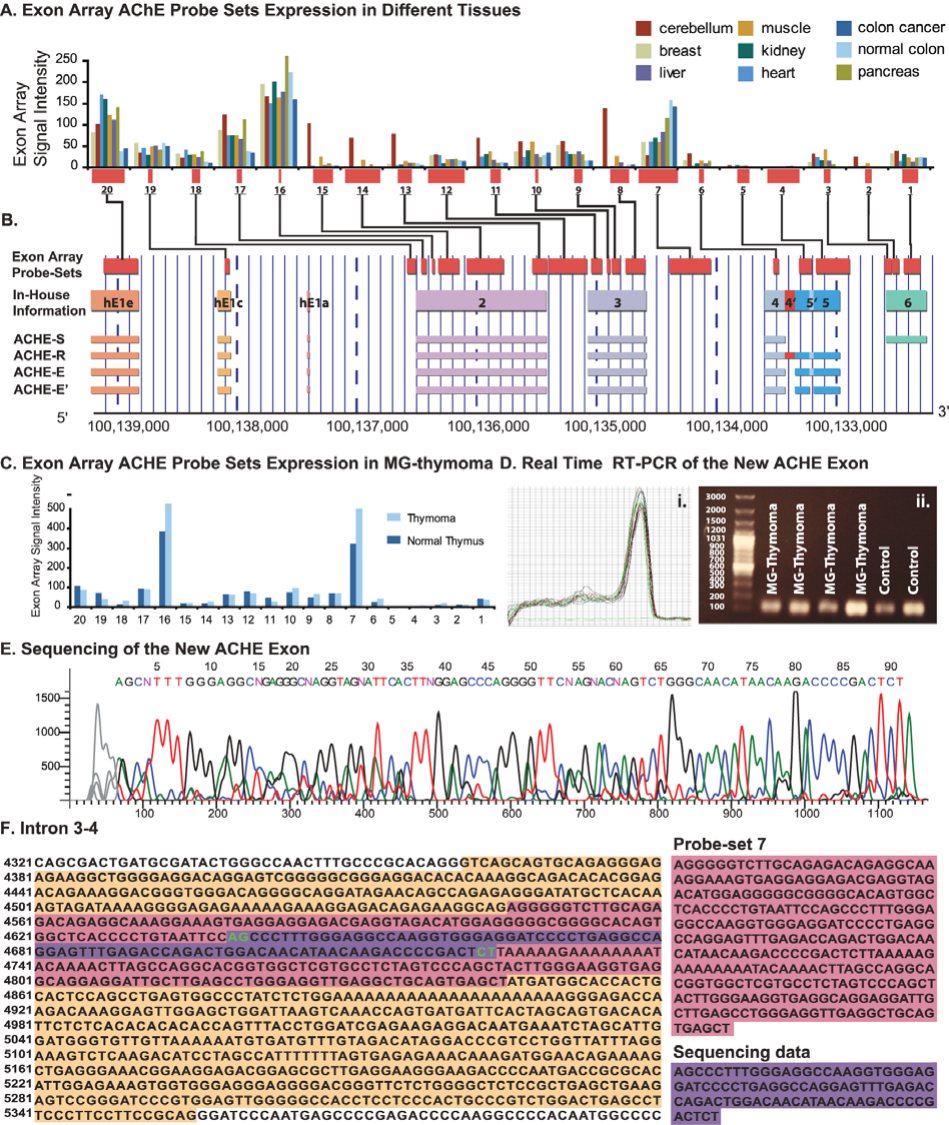
ACHE gene structure, alternative transcripts and protein products, and the discovery of a Novel ACHE exon. (A) Signal expression of ACHE exon array probe-sets in healthy pancreas, liver, muscle, kidney, heart, cerebellum and breast, colon cancer and healthy colon of Affymetrix exon array sample data sets, aligned to known isoforms annotations. Probe set number 7 (that lies within intron 3–4) and probe set number 16 (which is within constitutive exon 2) exhibit relatively high expression. (B) Chromosomal location of ACHE on the reverse strand of chromosome 7 q22. The different ACHE variants (R,S,E,E') are displayed in separate rows (data from [78]). Exon array probe sets (red) aligned to various variant annotation and prediction sources as given in the IGB tool are shown. RefSeq known annotations (as for Exon array genome version, 2003) are shown below them, in green and blue. (C) MG-thymoma expression of ACHE exon array probe sets. Intronic probe set number 12 and probe set 16 exhibits a clearly high expression in MG-thymoma. The signal intensity ACHE expression in MG-thymoma is significantly higher then in all other tissues, including colon cancer, (see (A) above). Real time (i) and RT-PCR (ii) expression of the novel exon between introns 3 and 4, on both MG-thymoma and healthy thymus samples. (E) Sequencing results of the RT-PCR product for the probe set between exons 3 and 4. (F) The sequence identified matches a portion of the target region, and contain AG-CT splice sites.

FISH for *AChE*-R protein variant indicated a significant increase in the number of stained cells in the MG-thymomic section compared to healthy thymus (Figure 5A). The largest constitutive exon of number 2 of *ACHE* (Figure 6A,C) showed pronounced expression compared to the other *ACHE* exons in exon array data sets from cerebellum, breast, liver, muscle, kidney, heart, pancreas and both normal and tumor colons (Figure 6A, gene structure under 6B). The mRNA levels of the constitutive exon 2 in the thymus, and yet more so in MG-thymoma tumors were higher than in all other examined tissues (Figure 6A–C) with mean signal intensity of 528.5 in MG-thymoma and 386.5 in healthy thymuses compared to mean signal intensity of 158.9 in colon cancer and 223.6 in healthy colon and 261.3 in healthy pancreas, the highest expression compared to all the other normal tissues. We found changes in two different categories to which *ACHE* belongs: *response to wounding* (BP) decreased according to the discrete approach compared to both *response to external stimulus* and *detection of bacteria* and compared to *response to external stimulus* (Table S3). *Muscle contraction* (BP) globally increased according to both the discrete approach (P = 0.02) and to the KS test (P = 0.01), compatible with our recent report [38]. Specifically, it changed compared to its direct parent terms: *organismal physiological process, taxis* (increased according to the continuous approach with a change in location, and decreased according to the discrete approach) and *establishment of localization* (increases and decreased according to the discrete approach).

In-depth analysis of the expression levels of all *ACHE* probe-sets (core, extended and full) in MG-thymoma yielded two potentially new exons. These *ACHE* exons also appeared in exon array data sets from colon cancer and multiple healthy tissues [10], [20]. One of the predicted *ACHE* exons (located in the intron between the constitutive exon 3 and the alternative exon 4) showed values close to those of the constitutive exon 2 (Figure 6A and 6C). Real-time and quantitative RT-PCR analyses of this exon confirmed expression (Figure 6D), and sequencing of the amplified intronic region validated its identity (Figure 6E and 6F). Repeat Masker [53] demonstrated multiple ALu repeats in the intron where this exon lies (intron 2–3, Text S4), suggesting ALu exonization [54], which may lead to a prematurely terminated or smaller *ACHE* product.

### Linking MG-thymoma exon array expression changes with proteomic analysis

**Figure 7:**
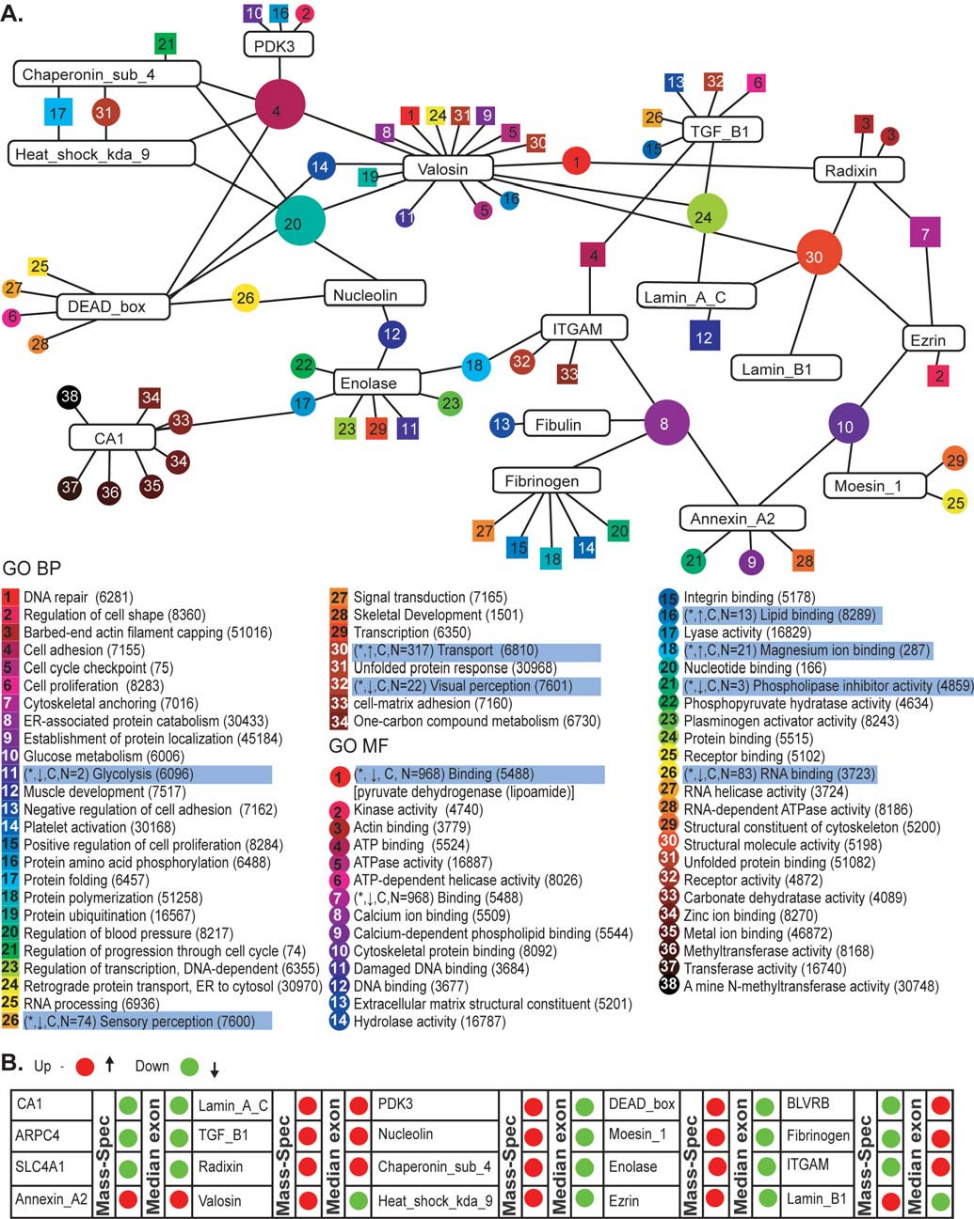
Proteomic Analysis Combined with Exon Array Data Indicates Possible Alternative Splicing and Overlaps with Changed GO Categories. Functional MF (circles) and BP (rectangles) GO term networks of selected changed proteins. The relative shape size (i.e circles and rectangles) indicates the number of connected proteins. Note the complex connections between proteins sharing common GO categories. Some of these categories changed according to the continuous exon array analysis as well. Comparison between protein and gene levels for proteins detected as changed using 1D MS analysis. Ninety-one changed proteins were detected using K-means clustering on the peptide signals information (supplementary methods). Relative signals for selected proteins were calculated from the mean number of peptides detected in MG-thymoma and healthy Thymus samples, and compared to the median exonic expression level of the corresponding genes as detected by the exon arrays. In some, both decreased, in some both increased and in the rest, opposite changes occurred.

The number of proteins expressed in mammalian tissues far exceeds that of the corresponding transcripts, likely due to alternative splicing [7], which expands the proteome by several orders of magnitude [55]. To link exon array data to protein products, we used MS of proteins from tested tissues, followed by functional annotation of the detected categories. The procedure is described under supplementary material[56]. Peptide sequencing obtained from 1D gel analysis of MG-thymoma and control thymuses (DataSet S1) yielded 91 proteins showing MG-thymoma-associated changes. These were divided into four groups using K-Means implementation according to fold changes between MG-thymoma and controls. Of these, about half demonstrated peptide sequences potentially belonging to more than one isoform, corroborating the exon array indications for alternative splicing. These included annexin A2, fibrinogen, chaperonin, heat shock protein, lamin A/C *and* DEAD box polypeptide 17, to name a few (Figure 7A and B).

Using the number of detected peptides as protein signals, we compared protein changes in the exon array and MS data. Many of the detected proteins shared MF and BP GO terms identified in our arrays (for example, both enolase and nucleolin share the *muscle development* category), and overlapping changes were observed in *binding* (Figure 7A). Thus, valosin-containing protein *(*VCP, marked as Valosin in Figure 7 *)* increased, both in the MS and according to the exon array analysis (Figure 7B). VCP contributes to ATP-dependent cellular processes and is required for the survival of breast cancer cells [57]. The ezrin, radixin and moesin proteins, involved in intracellular anchoring of cell membrane proteins to the cytoskeleton [58], were detected by both the MG-thymoma MS samples and the exon arrays (Figure 7B). MG-thymoma radixin median exonic change increased but ezrin and moesin decreased, reflecting specific regulation for each member of this protein family. Inversely, fibrinogen was only observed in control samples, perhaps indicating its de-stabilization in the tumors.

Taken together, the RT-PCR, FISH and MS validation tests were all supportive of the exon array findings of massive changes in alternative splicing, in spite of the inherent limitations in each of these methods (e.g. the requirement for tissue preservation, variable primer efficiencies and limited resolution power of peptide sequencing).

## Discussion

We combined exon Arrays with *ad-hoc* and *post-hoc* statistics, in-depth analysis of key modified transcripts and multi-leveled validation tests to identify a hyper-alternative splicing signature for MG-thymomas. Our findings highlighted hyper-splicing in tumor-related, immune function and muscle-specific transcripts with distinct patterns from those of colon cancer or healthy thymus and included parent-child relationships in the GO hierarchy [59], [60], which specifically highlighted biologically significant categories and transcripts. Comparison to other available microarray analysis techniques [61] demonstrated higher detection rate and greater accuracy of our approach. Importantly, *ASF/SF2* and *SC35*, previously shown as proto-oncogens [62] exhibited highest mRNA expression levels in the thymus then other tissues yet decreased in MG-thymoma compared to healthy thymus at both the protein and mRNA levels. Changes in the expression of these proteins can affect the alternative splicing of an undefined number of cellular transcripts and might account for some of the known splicing changes in cancer [63]–[65]. Our findings thus suggest aberrant tissue- and cancer-specific decline in alternative splicing, accompanied by alternative hyper-splicing. Specific changes related to MG-thymoma included *HLA-DRB1*, associated with a haplotype predictive of MG susceptibility in female [66] with specific gene variants. Up until now, the association between MG-thymoma to MHC haplotypes spanned a thymoma-associated class II allele on the DQB1 haplotype [67]. Also, B7, B8 (in early MG [68], [69]), DR3 (in early MG [67]), DR1, protective in MG [67] and the DR2 allele [15] all showed associations to MG. Our findings are hence indicative of alternative HLA-DRB transcript(s) as being involved.

We found general increases in muscle-related GO terms and increased myosin levels (both mRNA and protein). The mRNA increase was significant both in *MYH14* and in *MYH10*. A possible splice shift observed in *MYH14* gene, as compared to decrease of it mRNA in colon cancer. Indeed, antibodies against structural muscle proteins, among them *myosin,* were detected by others in the sera of MG patients [45], and myosin mutations are associated with colon cancer [70], [71].

For cancer to metastasize from a primary tumor, the extracellular matrix-physiological barrier whose primary structural constituent is collagen-must be degraded to allow the passage of tumor cells. Collagen is further involved in the immune response to metastasis [72]. In the collagen *COL1A1* gene, we detected up-regulation in both MG-thymoma and colon cancer. Another collagen important for cartilage collagen formation and for organization of the extracellular matrix [73], *COL11A1,* was up-regulated in MG-thymoma, perhaps reflecting a compensation mechanism(s) over the degradation of its protein product. The diverse expression levels of core exon probe sets in *COL11A1* between colon cancer and MG-thymoma tumors may unveil many unknown smaller transcripts, relevant to different types of cancer. Currently, most of cancer research is conducted using standard 3′ arrays, which are limited in their discovery scope. Our study highlights potential benefits from using high-resolution arrays in the study of cancer. Importantly, *type I collagen* reduces *DPT* mRNA levels [74], and *DPT* was down-regulated both in MG-thymoma and in colon cancer, suggesting inter-relationship between the observed changes.

Other MG-thyoma increased transcripts included the latrophilin *LPHN3*, which may function in both cell adhesion and signal transduction, processes that are both essential in tumorigenesis [75]. Also, *IRX2*, involved in the regulation of developmental processes via the WNT pathway and which is notably amplified in soft tissue sarcomas [76] was overexpressed. *CNTNAP2,* an exceptionally large gene which encompasses almost 1.5% of chromosome 7, and which functions in the vertebrate nervous system as both a cell adhesion and a receptor molecule [77] was increased as well. In most of these genes, we observed MG thymoma-associated alternative hyper-splicing events (Figure 2).

We selected the low-level expressed *ACHE* gene for challenging the resolution power of the Exon array technology. In both MG-thymoma and control thymuses, we detected both the predictable 3′ alternative splicing and a previously unknown exon between introns 3 and 4, validated using RT-PCR, real-time RT-PCR and sequencing on MG-thymoma samples. In-depth analysis of Affymetrix exon array data sets suggests ubiquitous expression of this novel exon, which likely originated by ALu exonization (AF8 and [54]). Further studies will be required for exploring the biological significance of this finding, for example under stress conditions or in neurodegenerative disease, where *ACHE* gene expression is altered [7], [78]. For example, inclusion of this exon may cause early terminated translation, yielding a shorter polypeptide with yet unknown stability and biochemical features. Parallel not yet known ALu -derived exons likely exist in numerous other genes[30], which calls for further exon array studies to establish the scope of this phenomenon at the post-transcriptional level.

MS and peptide sequencing of the MG-thymoma samples analyzed by exon arrays, followed by clustering of identified proteins enabled linking these data to median core gene-level changes and corresponding GO terms. Overall, the MS results were compatible with a complex pattern of regulation for specific exons, genes and protein products. Median core exonic changes thus corresponded well with tissue detection of relevant mRNAs and proteins, even for relatively rare products such as AChE*-R*. The MS approach enables simultaneous targeting of numerous proteins, but with limited resolution compared to that of exon expression arrays. Only major proteins are detectable, and these randomly break into peptides and may be falsely identified by the upper layer alignment software because only one or two peptides can be observed. Notably, MS-detected proteins sharing the same GO categories showed interactions with exon-array analysis results, suggesting networks relating to the examined disease. Additionally, proteins sharing one biological process are more likely to interact than proteins involved in distinct processes [79]. Also, dis-similarities between mRNA and protein changes may indicate measurement errors, post-translational events, distinct turn-over rates or alternative splicing. In contrast, FISH and immunohistochemistry are highly sensitive, albeit limited in scale, thus enabling identification of subtle yet specific mRNA and protein changes.

Combining exons, whole transcripts and functional analyses with protein data and available exon array data sets can add further support to exon array studies. In view of the complexity of alternative splicing processes, our finding of a clear hyper-splicing signature for MG-thymoma suggests physiological significance for this signature. This, in turn, supports a notion whereby such exon-array derived signatures can serve for diagnosis as well as for rational drug design. The information provided by exon array experiments thus expands our biological knowledge on known and new transcript variants, opening new potential avenues for research, diagnosis and therapeutics.

## Materials and Methods

### Human thymus tissue samples

Freshly discarded thymic fragments were obtained from immunologically healthy female and male patients undergoing corrective cardiovascular surgery or from MG patients undergoing prophylactic thymectomy at the Centre Chirurgical Marie Lannelongue (Le Plessis Robinson, France). All MG patients included in the study were treated with anticholinesterases but not with corticosteroids or immunosuppressors. Thymoma tissues were removed for therapeutic purpose and fragments of normal thymus tissues were removed to make the large vessels more accessible during cardiac surgery. Pathologists at the Centre Chirurgical Marie Lannelongue (Le Plessis Robinson, Paris, France) determined the classification of thymoma. The Institutional Review Board: Comité de Protection des Personnes de Kremlin-Bicêtre approved the use of human tissues based on verbal consents alone. Our IRB explained that written informed consents were not mandatory, since the tissues were initially harvested for therapeutic purposes and because all donors remained anonymous. Samples are described under Table S4.

### RNA isolation

RNA was extracted from frozen thymus samples using the RNeasy lipid tissue kit (Qiagen, Valencia, CA) as per manufacturer's instructions from healthy and pathological thymus samples. DNase treatment was applied to avoid DNA contamination. RNA integrity was confirmed by gel electrophoresis, and RNA concentration and purity were assessed spectrophotometrically.

### Human exon 1.0 ST microarray experiment

1 µg of total RNA from MG-thymoma (male, 44 and female, 53), control (male, 43 and female, 46) thymuses was labeled with the Affymetrix exon array whole transcripts sense targeting labeling assay and reagents, including r-RNA reduction and labeling with Streptavidin-phycoerithrin. Each sample was hybridized to a GeneChip® Exon 1.0 ST Array (Affymetrix, Santa Clara, CA, USA) according to manufacturer's instructions, and results scanned to create four .CEL files using Affymetrix GCS 3000 7G scanner and GeneChip Operating Software v. 1.3 to produce .CEL intensity files.

### Additional exon array data files and normalization

Human colon cancer and all other available tissues sample data sets of Affymetrix exon array in .CEL files were downloaded from Affymetrix web site (http://www.affymetrix.com/support/technical/sample_data/exon_array_data.affx).

The data was normalized using Affymetrix ExACT software to sketch normalize exon array data. Probes of all the probe sets (core, extended and full) were summarized using ExACT [80]. Gene-level iter-plier results are given under ST1. Each exon array probe-set is annotated in one of three possible levels: core, extended and full, according to the annotation source of the interrogated region. **Core** probe sets are supported by most reliable information, associated with full-length coding sequence mRNA evidence from RefSeq and GenBank records. **Extended** transcript clusters are based on cDNA evidence and include other human mRNA and EST sequences, ENSEMBL gene collections, synthetically mapped mRNAs from mouse and rat, mitoMap mitochondrial genes, microRNA registry genes, vegaGene and vegaPseudoGene records. **Full** Transcript Clusters are supported by gene and exon prediction algorithms including GeneID [81], GenScan [82], GenScanSubOpt [83], exoniphy [84], RNAGene [85], sgpGene [86] and Twinscan [87]. Each probe set contains several probes (typically 4) [10].

### Statistical data analysis and bioinformatics

#### Functional GO analysis

Mapping of transcript clusters to UniGene identifiers

Transcript cluster IDs were assigned to their corresponding UniGene clusters using transcript_annot file [80]. For each transcript, all the UniGenes that are included in its region were considered for further functional analysis.

Mapping of UniGene identifiers to corresponding GO terms

EASE [24] program served to identify all of the BP and MF GO terms that are represented on the human 1.0 ST Exon array. GO ontology files (www.geneontology.org) enabled defining of all the UniGenes (UG) associated with each represented GO term. Statistical tests found increases or decreases in expression using the discrete Fisher exact test with a 2-fold threshold, and continuous KS statistics, analyzing each tail separately.

Colon cancer exon array data set [20] functional analysis

20 samples of colon cancer and healthy colons [20] (N = 10 in each) were analyzed. For gene level analysis, we used iter-PLIER [80] gene level transcript signals of the colon cancer data set published by Affymetrix [20]. We calculated the mean log ratio, and coefficient variation (CV) score for each group (data available upon request). UniGene identifiers and their corresponding signals were analyzed using continuous and discrete GO analysis using KS and Fisher exact test, respectively.

Colon cancer arrays comparison using GO analyses

From colon cancer exon array data [20], changed genes (N = 159) obtained from Affymetrix quantile sketch normalization and ANOVA p-values and fold changes[20]. We applied EASE [24] to obtain enriched BP GO categories.

Enriched BP GO categories in the list of changed genes in colon cancer, using microarray analyses, obtained from Bush and coworkers [35], where ErmineJ [33] was used to analyze GO of 1975 differentially expressed probesets identified by the empirical Bayes HotellingT2 model [35]. GO analyses of protein maps obtained by 2D gel MS analysis of colon cancers were obtained from Patel and coworkers [37]. A list of changed BP GO categories using functional analysis of colon cancer using microarrays was obtained from Maglietta and coworkers [36]. Full results table will be given upon request.

MG-thymoma exon array data set functional analysis

UG identifier lists were extracted and then GO [88] annotations of these lists were obtained by EASE [24]. To obtain gene level signals, the median core exonic fold change of each UG cluster was calculated. UG identifiers and their corresponding signals were grouped into their corresponding GO terms, and analyzed using continuous and discrete GO analysis using KS and Fisher exact test, respectively, as compared with the global array population. Additionally, global term-to-parents analysis was conducted on the whole group of array-represented GO terms, to compare each term to each of its direct parent terms, and to its GO tree root (i.e MF/BP). This specific analysis was conducted using KS, Fisher exact, Kruskal-Wallis and variance tests.

MG-thymoma exon array permutated data set

To perform permutations, the median fold changes of the thymus samples were mixed twice. Then, log2 ratio between the mixed datasets was calculated. Continuous and discrete analyses were performed on the MG-thymoma permutated data (results under Supplementary Material). KS, variances and Kruskal-Wallis tests also served to examine specific changes in GO categories compared to their direct and indirect parent GO terms.

Database construction

A MySQL database was constructed and used to store exon array expression signals at the exon probe-set level, as well as annotation data from the analyzed samples, in corresponding tables, for efficient transcript-specific queries.

Other applications

An upper layer Java program was used to access specific transcripts information. To examine global exonic and gene-level signals at different gene fragments, a Visual Basic application was written. Matlab 7.1.0.246–R14 [89] used to perform all statistical analyses.

### Immunohistochemistry

Polyclonal antibodies for SC35, Myosin (Sigma, St. Louis, MI) and AS/SF2 (Zymed, San Francisco, CA) were used at 1∶100, 1∶100, 1∶20 dilutions, respectively. Sections were deparaffinized, permeabilized and incubated with 100 mM Glycine in PBS for 20 min at room temp, preblocked with buffer containing 5% donkey serum, 0.5% Tween20 in PBS (1 h, at room temp) and incubated with primary antibodies (overnight, at 4°C). Biotinylated antibodies were incubated with Cy3-conjugated streptavidin (Jackson ImmunoResearch Laboratories, West Grove PA, USA). Sections were coverslipped and analyzed by confocal microscopy using a Bio-Rad MRC-1024 scanhead (Hemel Hempsted, Hertfordshire, U.K.) equipped with a digital camera and Olympus FV-1000 confocal microscopes, using excitation and emission parameters suitable for Cy3. Four areas (54 μ^2^) were photographed from each section with *SC35* and *ASF/SF2* labeling and the mean density per area was quantified with the software package ImagePro4 (Media Cybernetics, Silver Spring, MD). Myosin immunohistochemistry was performed on 106μ^2^ sections and the same software was used to analyze the number of stained cells per area. Student's *t*-test was used to determine the statistical significance between healthy and MG-thymoma sections.

### Fluorescence In Situ Hybridization (FISH)

Deparaffinization and rehydration of the tissue, permeabilization by proteinase K and prehybridization were as detailed elsewhere [39]. Following prehybridization for 30 min, at 60°C with hybridization buffer (50% formamide, 750 mmol/L sodium chloride, 75 mmol/L sodium citrate at pH 4.5, 50 µg/mL heparin and 50 μg/mL tRNA), hybridization was performed for (2h, 52°C) with 1 µg/mL 5′-biotinylated, 2-O-methylated *ACHE* cRNA probe complementary to human AChE-R mRNA (Microsynth GMBH (Balgach, Switzerland)). Microscopy and data analysis involved four 106μ^2^ areas from each section and stained cell numbers per area were determined.

### Real-time RT-PCR

For each sample, 0.4 µg RNA was used for 20 µl cDNA synthesis using Promega RT-PCR kit (Promega, Madison, WI). Real-time RT-PCR was performed in triplicate for each sample using ABI prism 7900HT and SYBR green master mix (Applied Biosystems, Foster City, CA). ROX, a passive reference dye, was used for signal normalization across the plate. Primer sequences are described under Table S5. Annealing temperature was 60° C for all primers. Serial dilution of samples served to evaluate primers efficiency and the appropriate cDNA concentration that yields linear changes. Melting curve analysis and amplicon sequencing verified the end product and β-actin served as reference gene.

* Additional Materials and methods are under Text S5.

## Supporting Information

Figure S1 The number of GO categories presenting discrete and continuous changes in MG-thymoma by Venn diagrams. BP and MF categories that presented discrete (denoted as D) 2-fold change (dark gray) or continuous (denoted as C) change of median transcript exonic expression level using KS statistics (light gray) and both methods (intersection areas) as compared with the total population of UniGene clusters represented on the array. Note that in both MF and BP, more categories decreased than increased, and more categories showed change in the continuous approach than the discrete.
(0.14 MB TIF)

Figure S2 Tumor-specific Gene and Exon Level Expression Changes. Exon level probe sets showed a decrease fold change trend in MG-thymoma compared to healthy thymus (Ai). In colon cancer, an inverse increase trend appeared compared to healthy colon data (Aii). In both, the trend was conserved across all annotation levels-core, extended and full (Ai and Aii). Specifically, core level exons decreased in MG-thymoma compared to colon tumorgenesis events (Aiii). Median gene-level global exon array population exhibited decrease in MG-thymoma tissues compared to healthy thymuses, corresponding to exon-level changes (Aiv). The change was significant and differed from that of permutated populations (Figure S3). (B) The center 80% exons of all transcripts showed larger expression decrease than both 3′ and 5′ 10% portions (statistical information under Supplementary Material). (C) Focusing only at terminal probes, the 3′ of MG-thymoma samples exhibited decrease of the exons in the 3′ 60 bps, compared to the 600 bps region (low KS P-value <0.05), with a change in dispersion (variances test P-value = 0; exon data was included if at least half of it was within the tested transcript boundaries). (D) Exonic changes were slightly different between 3′ to the 5′ 10% genomic region of all transcripts. 3′ edge increased pronouncedly with changes both in location (i) and in the number of genes with changed exons (ii). (E) At the 5% genomic region, the 3′ edge regions decreased compared to the 5′, with a difference in location and dispersion (i) and the number of changed genes (ii). (F) The 2.5% fragment resolution revealed a striking difference in exonic change patterns between the 3′ to the 5′ edges, expressed in distributional tails, location and dispersion (i) as well as the number of changed genes (ii).
(1.17 MB TIF)

Figure S3 Comparison of total global median core exons change in MG-thymoma and colon cancer. A) The median core exonic gene level signal is shown for MG-thymoma and colon cancer, for all the UniGene clusters represented on the array. Generally, colon cancer (N = 10) increased compared to MG-thymoma changes (N = 4) significantly (high KS P-value = 0). MG-thymoma and colon cancer samples were compared to matching healthy samples accordingly, and then to one another. B) (i) Median core exonic gene level signal, for MG-thymoma compared to healthy thymus samples (blue), with permutated patients and healthy samples changes (green and red). (ii) Median core exonic gene level signal, for colon cancer compared to healthy colon samples (blue), with permutated patients and healthy samples changes (green and red).
(0.63 MB TIF)

Figure S4 Comparison of median expression change in MG-thymoma 3′ 60 to 600 base pairs. Median exonic change of all array transcripts, was considerably smaller within the 3′ 60 nucleotides (N = 2,131) then in the 3′ 600 bps (N = 16,318). Statistical information under Supplementary Material.
(0.36 MB TIF)

Figure S5 Comparison between expression differences in various gene edges between the 3′ to 5′ of genes. A. MG-thymoma compared to healthy thymus samples (i)Median Exonic change of 3′ compared to 5′ 10% edge fragments of all array transcripts. There was a slight increase (CDF plot) in the 5′ Exonic change (high KS<0.05) with a change in location and dispersion. (ii) Median Exonic change of 3′ compared to 5′ 5% edge fragments of all array transcripts. There was a slight decrease (CDF plot) in the 5′ Exonic change (high KS<0.05) with a change in location and dispersion. (iii) Median Exonic change of 3′ compared to 5′ 2.5% edge fragments of all array transcripts. A striking difference between edges is observed, at both distributional tails (high and low KS P-values = 0), and both dispersion and location. (iv) In the 5′ edge, no change between median exonic changes observed between 10%, 5% and 2.5% of all the transcripts. B. Colon cancer compared to healthy colon samples Median Exonic change of 3′ compared to 5′ 2.5% edge fragments of all array transcripts. There was a decrease (CDF plot, low KS P-value = 0.05) in the 5′ Exonic change compared to the 3′ edge.
(0.56 MB TIF)

Figure S6 Specific GO category change compared to its parent terms. The change in RNA binding (N = 83) category compared to both direct and indirect GO parents. The blue and red bars indicate UniGenes that decrease and increased more then 2-fold, accordingly. (i) RNA binding decreased (low KS P-value <0.05) compared to the global parent term, MF (N = 21,047). (ii) RNA binding decreased (low KS P-value <0.05) also compared to its direct parent term, nucleic acid binding (N = 280).
(0.60 MB TIF)

Figure S7 Median core exons change of ASF/SF2, SC35, Myosins and AChE-R. The median core exonic log fold change as compared between MG-thymoma to healthy thymus samples, for the validated genes. Myosin (MYH10), lymphocyte-specific myosin (MYLC2PL) and AChE-R increased, whereas SC-35 and ASF/SF2 decreased.
(0.05 MB TIF)

Text S1 Functional Analysis of Colon Cancer Data Set Exhibits Large Overlaps with Other Colon Cancer Data Sets. Results of comparisons between our continuous and discrete ad-hoc functional GO statistical analysis to other ad- and post- hoc functional GO analyses on colon cancer microarray and proteomic data sets.
(0.10 MB DOC)

Text S2 Global Exons Probe Sets Changes in Different Gene Portions of MG-Thymoma and Colon Cancer. Statistical results of the comparisons between different gene edges (3′ and 5′ regions) expression changes between MG-thymoma to healthy thymuses, and differs from permutated MG-thymoma data set.
(0.03 MB DOC)

Text S4 The Intron in which the New ACHE Exon is Located is Enriched with ALu Repeats. Results of repeat masker [53] on the constitutive ACHE exons number 2 and 3, intron 2–3 and intron 3–4 (where the new exon is located).
(0.04 MB DOC)

Text S3 Core Probe Sets Normalized Signals Intensity of Detected MG-Thymoma genes. Expression signals intensity of core probe sets of the MG-thymoma genes detected by the specific term to parent ad-hoc approach combined with fold change threshold are given for MG-thymoma and healthy thymuses.
(0.09 MB DOC)

Text S5 Additional materials and methods
(0.04 MB DOC)

DataSet S1 1D-Gel Mass Spectrometry Proteomic Analysis Results on the MG-Thymoma and Healthy Thymus Samples. All the identified peptides are given, with the corresponding proteins identified by clustering analysis and NCBI database [4]
(1.72 MB XLS)

Table S1 Ad-hoc Global Functional BP and MF GO Analysis of Exon Array Colon Cancer Data. Results of Affymetrix exon array colon cancer data set [20] are given for MF and BP. The columns of the Excel table are as indicated: the name of GO term, its GO ID, p-values of the continuous method (KS-P high tail test), testing the hypothesis that the tested distribution contains larger values than the global distribution, of Fisher exact test (Fishex INC, the high tail test) using the hypergeometric distriution (i.e, the discrete method), testing the hypothesis that the tested distribution contains larger than expected number of increased (INC) genes (using a 2-fold cutoff), the KS-P (low tail) testing the hypothesis that the distribution contains smaller values than those observed with the global distribution and Fishex (DEC) for testing the hypothesis that the testes distribution contain larger than expected number of decreased (DEC) genes (using a 2-fold cutoff). N is The number of transcripts associated with the GO term, KS accur (KS accuracy) is a number indicating the reliability of the KS test. The p-value is most accurate when KS accur >4. Additionaly, the percent of transcripts changed above the threshold is given for each category (named % changed genes), and the number of genes changed more then the threshold (named #changed genes). The red and blue cells in the tables indicate the GO terms that significantly increased or decreased, respectively (at significance level of p-value <0.05).
(0.62 MB XLS)

Table S2 Ad-hoc Global Functional BP and MF GO Analysis of Exon Array MG-Thymoma Data Set. Results of Affymetrix exon array MG-thymoma data set are given for BP and MF. The columns of the Excel table are as follows: Name (GO term title) ,GO ID, KS-P (high), Fishex (INC),KS-P (low),Fishex (DEC), KS-P (high), percent changed genes and number of changed genes. The red and blue cells in the tables indicate the GO terms that significantly increased or decreased, respectively (p<0.05). Detailed description of each column is given under S1 description.
(0.37 MB XLS)

Table S3 Specific, Term-To-Parent Functional GO Analysis Results on MG-Thymoma Data Set. Results of hierarchical comparison between GO terms to their direct and indirect (BP/MF) parent GO terms in MG-thymoma exon array data set are given for all the terms represented on Affymetrix exon 1.0 S_T array. The columns of the Excel table are: Name (of GO term), GO ID (of the term), Parent name (the name and GO ID of the compared parent, KS-P (high): p-value for the one tailed KS test (ie, the continuous method), testing the hypothesis that the tested distribution contains larger values than the global distribution, Fishex (INC): p-value for the Fisher exact test using the hypergeometric distribution (ie, the discrete method), testing the hypothesis that the tested distribution contains a larger than expected number of increased (INC) genes (using a 2-fold cutoff), KS-P (low)-a p-value for the one tailed KS test (ie, the continuous method), testing the hypothesis that the tested distribution contains smaller values than those observed with the global distribution, Fishex (DEC): a p-value for the Fisher exact test using the hypergeometric distribution (ie, the discrete methods), testing the hypothesis that the testes distribution contain larger than expected number of decreased (DEC) genes (using a 2-fold cutoff), N-the number of transcripts associated with the GO term, KS accur (KS accuracy)-a number indicating the reliability of the KS test. The p- value is most accurate when KS accur >4.VAR: P value for variances (VAR) test indicating a change in dispersion and of Kruskal Wallis (KW) test indicate a change in location. The percent of transcripts that changed more than the threshold (both increased and decreased) of all the GO term transcripts (% change genes), the number of changed genes (# changed genes)-the number of transcripts that changed more than the threshold (both increased and decreased) out of all the GO term transcripts. The red and blue cells in the tables indicate the GO terms that significantly increased or decreased, respectively, at significance level of 0.05.
(5.76 MB XLS)

Table S4 Samples Description of MG-Thymoma and Healthy Thymus. Detailed description of the MG-thymoma and healthy thymus data set.
(0.03 MB XLS)

Table S5 PCR Primers for ACHE New Exon. PCR primers design obtained from Primer3 software (www.frodo.wi.mit.edu/) and were used for both gel electrophoration of RT-PCR and for Real-Time RT-PCR.
(0.03 MB XLS)
