# Supplementary figures and images for: Identifying Alternative Hyper-Splicing Signatures in MG-Thymoma by Exon Arrays
Source: PLoS One. 2008 Jun 11;3(6):e2392. doi: 10.1371/journal.pone.0002392 (PMC2409220; doi:10.1371/journal.pone.0002392)

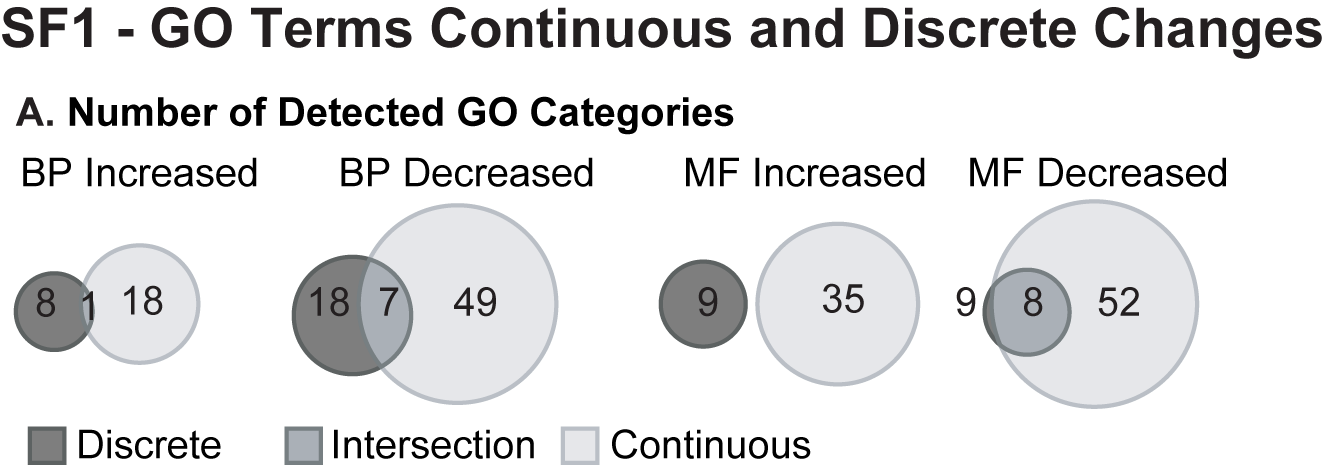

Supplement: Figure S1 — The number of GO categories presenting discrete and continuous changes in MG-thymoma by Venn diagrams. BP and MF categories that presented discrete (denoted as D) 2-fold change (dark gray) or continuous (denoted as C) change of median transcript exonic expression level using KS statistics (light gray) and both methods (intersection areas) as compared with the total population of UniGene clusters represented on the array. Note that in both MF and BP, more categories decreased than increased, and more categories showed change in the continuous approach than the discrete. (0.14 MB TIF) [file pone.0002392.s001.tif]

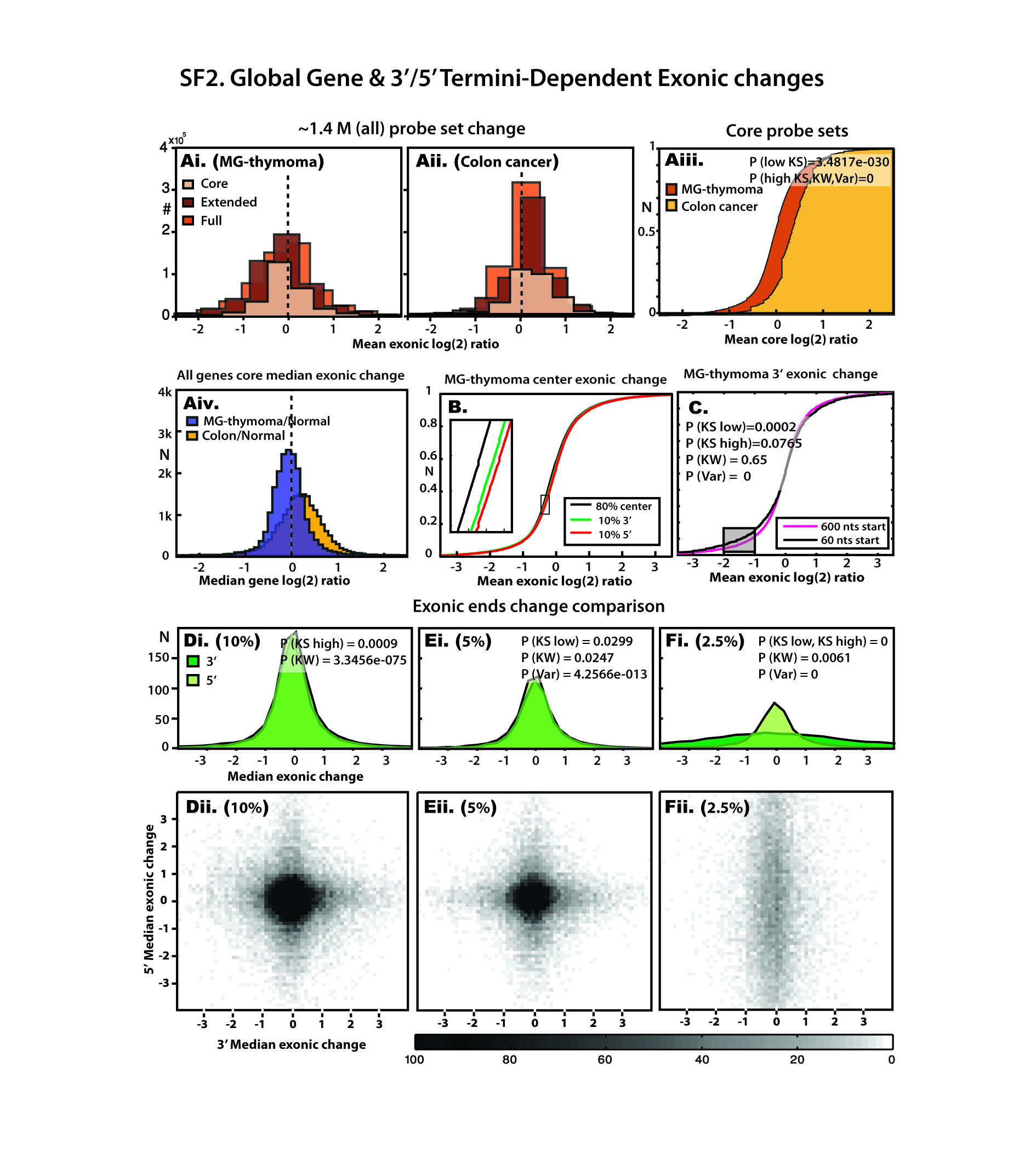

Supplement: Figure S2 — Tumor-specific Gene and Exon Level Expression Changes. Exon level probe sets showed a decrease fold change trend in MG-thymoma compared to healthy thymus (Ai). In colon cancer, an inverse increase trend appeared compared to healthy colon data (Aii). In both, the trend was conserved across all annotation levels-core, extended and full (Ai and Aii). Specifically, core level exons decreased in MG-thymoma compared to colon tumorgenesis events (Aiii). Median gene-level global exon array population exhibited decrease in MG-thymoma tissues compared to healthy thymuses, corresponding to exon-level changes (Aiv). The change was significant and differed from that of permutated populations (Figure S3). (B) The center 80% exons of all transcripts showed larger expression decrease than both 3′ and 5′ 10% portions (statistical information under Supplementary Material). (C) Focusing only at terminal probes, the 3′ of MG-thymoma samples exhibited decrease of the exons in the 3′ 60 bps, compared to the 600 bps region (low KS P-value <0.05), with a change in dispersion (variances test P-value = 0; exon data was included if at least half of it was within the tested transcript boundaries). (D) Exonic changes were slightly different between 3′ to the 5′ 10% genomic region of all transcripts. 3′ edge increased pronouncedly with changes both in location (i) and in the number of genes with changed exons (ii). (E) At the 5% genomic region, the 3′ edge regions decreased compared to the 5′, with a difference in location and dispersion (i) and the number of changed genes (ii). (F) The 2.5% fragment resolution revealed a striking difference in exonic change patterns between the 3′ to the 5′ edges, expressed in distributional tails, location and dispersion (i) as well as the number of changed genes (ii). (1.17 MB TIF) [file pone.0002392.s002.tif]

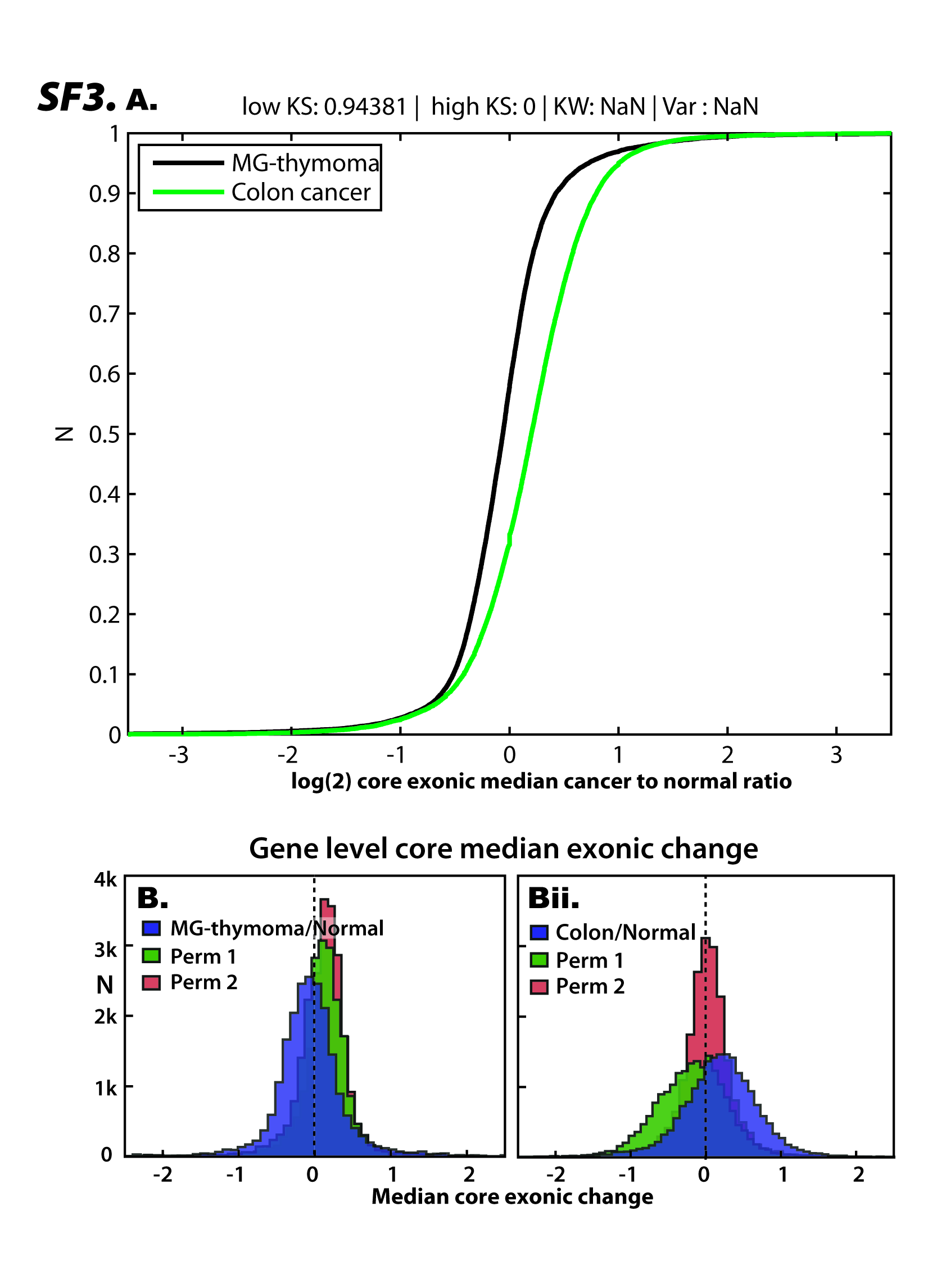

Supplement: Figure S3 — Comparison of total global median core exons change in MG-thymoma and colon cancer. A) The median core exonic gene level signal is shown for MG-thymoma and colon cancer, for all the UniGene clusters represented on the array. Generally, colon cancer (N = 10) increased compared to MG-thymoma changes (N = 4) significantly (high KS P-value = 0). MG-thymoma and colon cancer samples were compared to matching healthy samples accordingly, and then to one another. B) (i) Median core exonic gene level signal, for MG-thymoma compared to healthy thymus samples (blue), with permutated patients and healthy samples changes (green and red). (ii) Median core exonic gene level signal, for colon cancer compared to healthy colon samples (blue), with permutated patients and healthy samples changes (green and red). (0.63 MB TIF) [file pone.0002392.s003.tif]

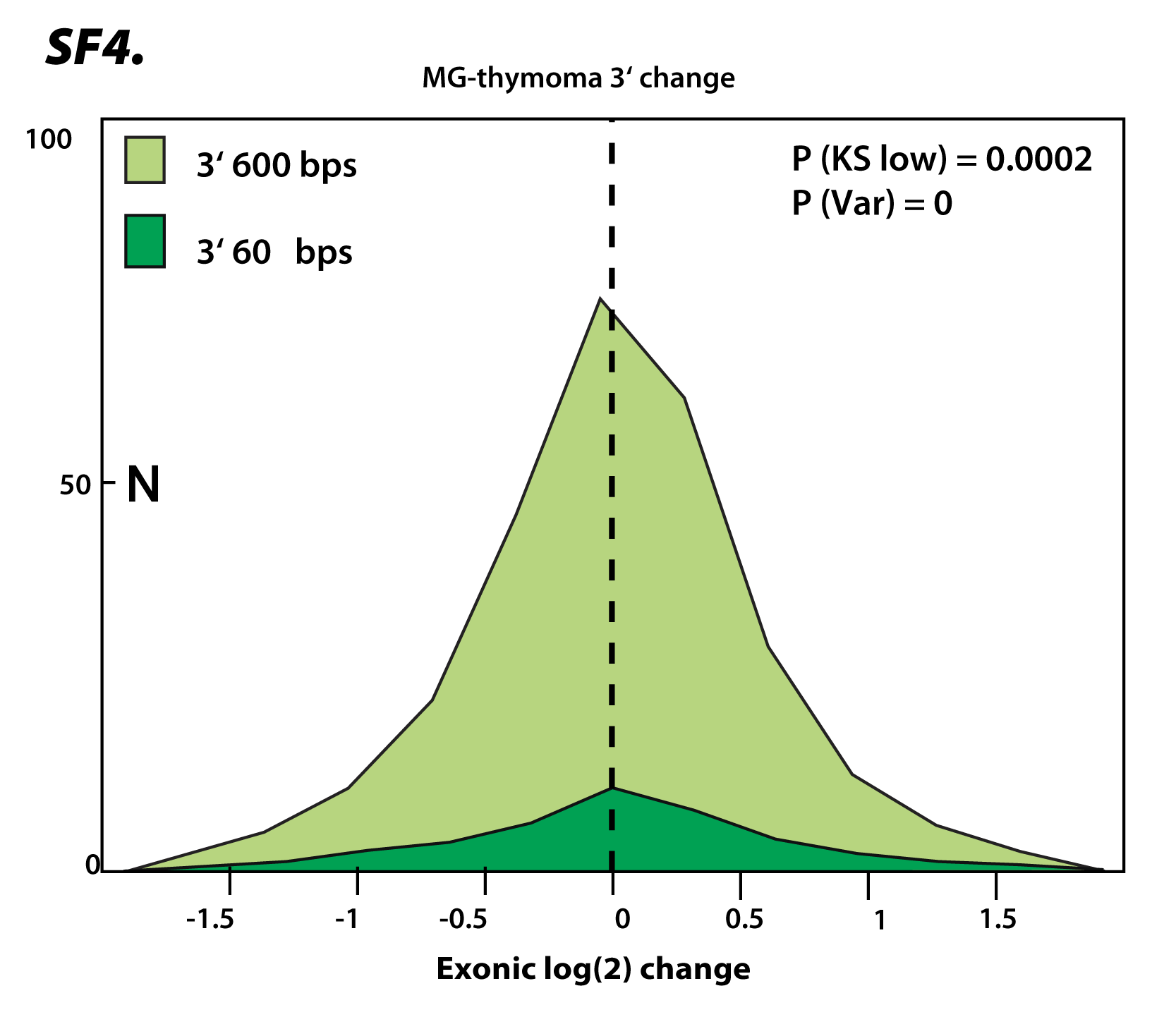

Supplement: Figure S4 — Comparison of median expression change in MG-thymoma 3′ 60 to 600 base pairs. Median exonic change of all array transcripts, was considerably smaller within the 3′ 60 nucleotides (N = 2,131) then in the 3′ 600 bps (N = 16,318). Statistical information under Supplementary Material. (0.36 MB TIF) [file pone.0002392.s004.tif]

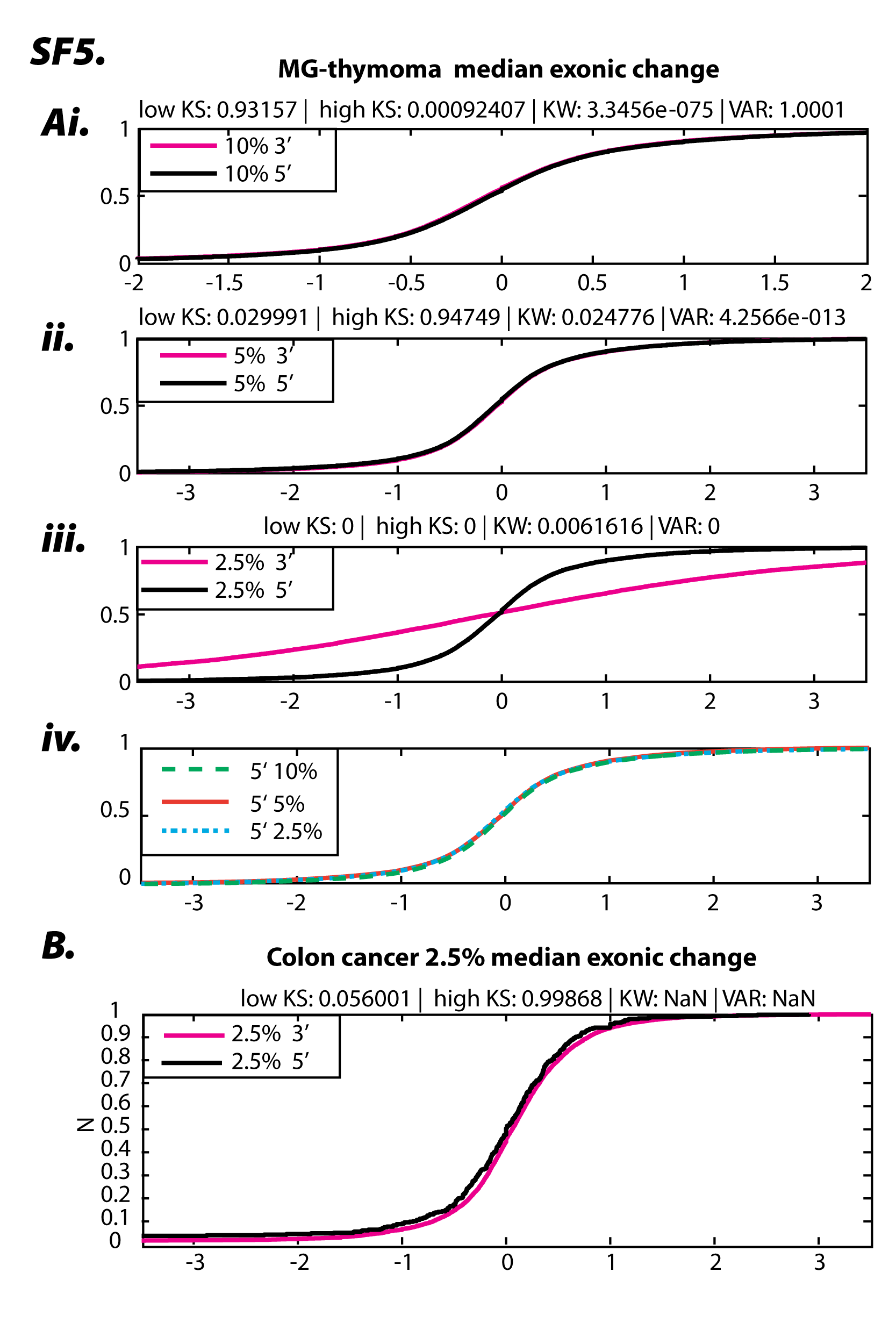

Supplement: Figure S5 — Comparison between expression differences in various gene edges between the 3′ to 5′ of genes. A. MG-thymoma compared to healthy thymus samples (i)Median Exonic change of 3′ compared to 5′ 10% edge fragments of all array transcripts. There was a slight increase (CDF plot) in the 5′ Exonic change (high KS<0.05) with a change in location and dispersion. (ii) Median Exonic change of 3′ compared to 5′ 5% edge fragments of all array transcripts. There was a slight decrease (CDF plot) in the 5′ Exonic change (high KS<0.05) with a change in location and dispersion. (iii) Median Exonic change of 3′ compared to 5′ 2.5% edge fragments of all array transcripts. A striking difference between edges is observed, at both distributional tails (high and low KS P-values = 0), and both dispersion and location. (iv) In the 5′ edge, no change between median exonic changes observed between 10%, 5% and 2.5% of all the transcripts. B. Colon cancer compared to healthy colon samples Median Exonic change of 3′ compared to 5′ 2.5% edge fragments of all array transcripts. There was a decrease (CDF plot, low KS P-value = 0.05) in the 5′ Exonic change compared to the 3′ edge. (0.56 MB TIF) [file pone.0002392.s005.tif]

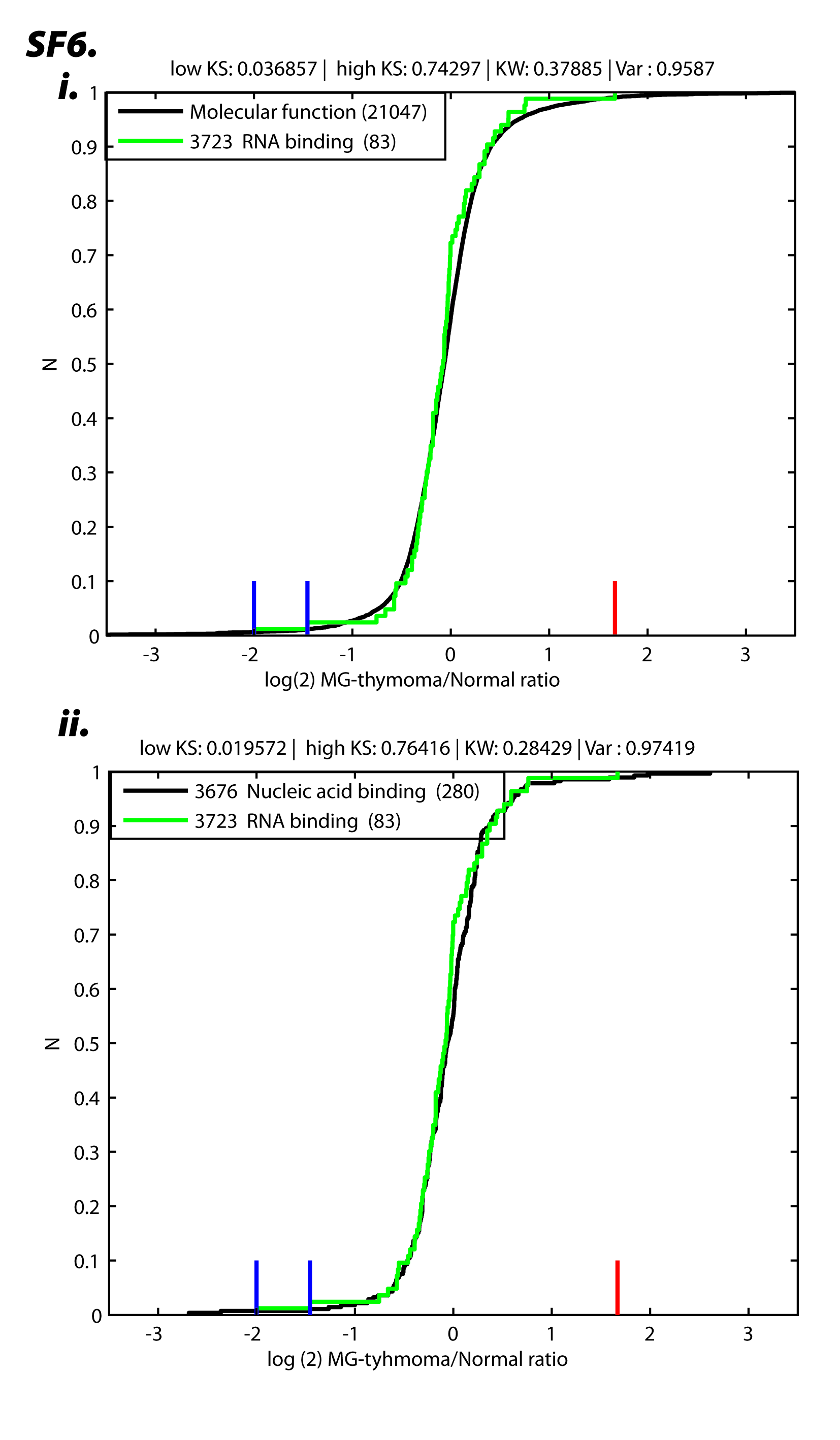

Supplement: Figure S6 — Specific GO category change compared to its parent terms. The change in RNA binding (N = 83) category compared to both direct and indirect GO parents. The blue and red bars indicate UniGenes that decrease and increased more then 2-fold, accordingly. (i) RNA binding decreased (low KS P-value <0.05) compared to the global parent term, MF (N = 21,047). (ii) RNA binding decreased (low KS P-value <0.05) also compared to its direct parent term, nucleic acid binding (N = 280). (0.60 MB TIF) [file pone.0002392.s006.tif]

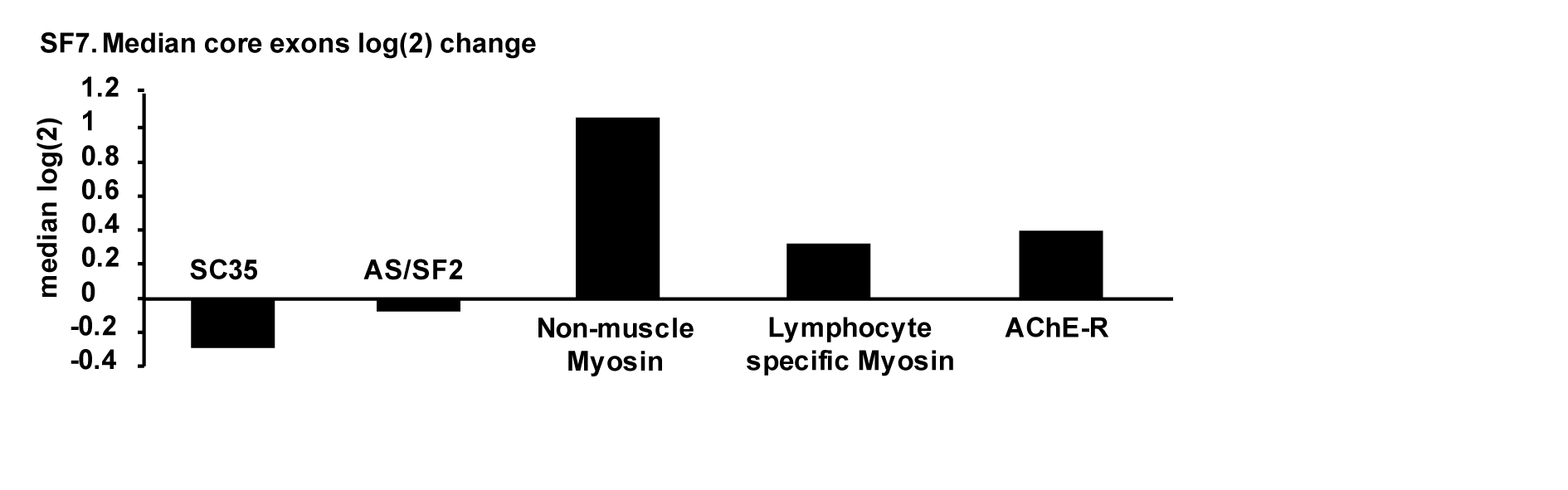

Supplement: Figure S7 — Median core exons change of ASF/SF2, SC35, Myosins and AChE-R. The median core exonic log fold change as compared between MG-thymoma to healthy thymus samples, for the validated genes. Myosin (MYH10), lymphocyte-specific myosin (MYLC2PL) and AChE-R increased, whereas SC-35 and ASF/SF2 decreased. (0.05 MB TIF) [file pone.0002392.s007.tif]
