# Supplementary material for: Identifying Alternative Hyper-Splicing Signatures in MG-Thymoma by Exon Arrays
Source: PLoS One. 2008 Jun 11;3(6):e2392. doi: 10.1371/journal.pone.0002392 (PMC2409220; doi:10.1371/journal.pone.0002392)
Supplement: Text S1 — Functional Analysis of Colon Cancer Data Set Exhibits Large Overlaps with Other Colon Cancer Data Sets. Results of comparisons between our continuous and discrete ad-hoc functional GO statistical analysis to other ad- and post- hoc functional GO analyses on colon cancer microarray and proteomic data sets. (0.10 MB DOC) [file pone.0002392.s008.doc]

# Text S1

We combined our ad-hoc with post-hoc methods on iter-PLIER gene-level summaries of Affymetrix colon cancer data set1 to examine our approach . This yielded a considerably wider spectrum of detected changes than in either of these approaches alone. To compare our ad-hoc exon array functional method to other, gene-level (i.e post-hoc) methods applied to colon cancer data sets^1,2,3,4^, we identified GO categories enriched in colon cancer by (1) ad-hoc approach on conventional 3’ arrays2 and (2) several post-hoc methods using exon or conventional arrays and proteomic (MS) data analyses^1, 3, 4^. We found large fractions of co-detected categories with all other methods (Table 1), yet with considerably larger detection efficiency in our ad-hoc functional method.

Continuous and discrete data analyses reveal colon cancer-affected GO terms

To establish our approach we initially analyzed Affymetrix the colon cancer data set1 by using the Affymetrix iter-PLIER gene-level signal estimates^1^ which provide exon array transcript signals. The transcript region definition of the exon arrays may refer to several genes^2^; therefore, we mapped the transcripts to UniGene clusters, and then found their current GO categories using EASE5 (detailed description under Materials and Methods (Additional File AF1)). We tested the categories using both discrete (D) (using a 2-fold change cutoff) and continuous (C) statistical tests. *Ad-hoc* functional analysis results of the Affymetrix colon cancer data set are given under Supplementary Table ST2 as BP and MF ontologies. Among the changed categories, we found GO terms clearly relevant to tumor development. For example - *angiogenesis* (BP, N=20, ↓ C D, ↑ D, P<0.05 where up and down arrows note increase and decrease, respectively), and its ancest*or* - *blood vessel development* (BP, N=21, ↓ C D, ↑ D, P<0.05) which decreased according to the threshold based method and increased according to the continuous approach. Among the categories that exhibited a change, we found for example *damaged DNA binding* (MF, N=58, ↑ C D) and *regulation of signal transduction* (MF, N=9, ↑ , C).

Compatibility of colon cancer functional analysis with other array analysis tools

To challenge the quality of our approach and to estimate its detection power, we compared our global functional *ad-hoc* analysis results (i.e term-to global array population) on the Affymetrix exon array colon cancer data set^1^ to several ad- and *post-hoc* functional analyses of other colon cancer array datasets^1-4^. Briefly, we compared the following functional results:

1. By applying EASE5 on the full list of changed genes in the Affymetrix colon cancer exon array data1 (N=159 genes), we obtained a *post-hoc* list of enriched BP categories in the Affymetrix colon cancer exon array data1.
2. Colon cancer standard 3' microarray probe-sets revealed 1975 differentially expressed transcript and 111 enriched BP GO categories by the empirical Bayes HotellingT2 model3.
3. Various statistical methods on colon cancer data from standard 3' microarrays yielded an *ad-hoc* list of changed BP GO categories2.
4. Proteomic data obtained by MS analysis of colon cancers proteins following 2D gel separation4 provided 34 enriched BP GO categories .

We compared the BP categories detected in each of these methods to our functional analysis of BP terms at significance level of p-values of 0.05 and 0.01 (Table 1 and ST2).

Among the compared methods, the largest overlap with co-detected categories emerged with the proteomic MS post-hoc GO analysis (97.06%, Table 1)^4^. However, while MS post-hoc functional analysis detected the smallest number of categories, our ad0hoc integrative method detected the largest number of changed BP categories at significance level of p<0.05 (379, VS. 34, Table 1). At this cutoff, 44.59% of the detected BP categories were also detected by the other compared ad- and post- hoc studies (Table 1). At a more conservative acceptance cutoff of p<0.01, an even larger overlap (56.63%) was observed with data from other methods (Table 1). The closest method of those compared (that showed 56.76% overlap of detected categories3 (Table 1), but once again the number of detected categories by our method at p<0.01 was significantly larger (196 compared to 111). Of the categories that were detected by more than one method, several BP categories were highlighted by being detected by four, or all compared methods. Those included categories related to cell cycle such as M-phase, mitotic cell cycle, cell cycle check point and regulation of mitosis. Additional tumorigenesis-relevant functions that were over-represented in the compared data sets are metabolism (of both lipids and RNA), morphogenesis and localization of proteins. Hence, continuous and discrete statistical analyses of exon array gene-level data can identify biologically relevant categories in gene expression data, and enlarge the number of detected categories with a high level of reliability. Next, we asked how many of the categories that were detected by more than one method, were detected by each of these methods. Strikingly, 99.52% of the categories that were detected by at least two of the compared methods, were detected by our ad-hoc combined of post-hoc analysis on the exon array colon cancer data set^1^, and appear in the gene list found in this data set1 by the post-hoc analysis. Importantly, our combination yielded 100% of the categories detected by at least 3 methods, and by all methods (Table 1). Thus, combination of continuous and discrete statistical analyses of exon array data sets with gene list methods of the colon cancer data set^1^ emerged as a powerful approach for detection of both subtle and rigorous expression changes.

Table 1. Colon cancer post- and ad-hoc microarray analysis comparisons

| **Method** | **BP categories** | **Overlap with any other method (#,%)** | **Categories common to**  **>=2** | **Categories common to**  **>=3** | **Categories common to**  **4** | **Ref.** |
| --- | --- | --- | --- | --- | --- | --- |
| GO permutations | 86 | 23, (26.74%) | 11.11% | 10.74% | 31.82% | Maglietta^2^(ad-hoc) |
| protein list | 34 | 33, **(97.06%**) | 15.94% | 11.57% | 27.27% | Patel^4^ (post-hoc, proteomics) |
| gene list (FC) | 111 | 63,(56.76%) | 30.43% | 32.23% | 81.82% | Bush^3^ (post-hoc) |
| GO terms, D&C | 196 | 111,**(56.63%)** | 53.62% | 91.74% | 95.45% | Soreq on Gardina^1^  (ad-hoc, P<0.01) |
| gene list (AS) | 373 | 164,(43.97%) | 79.23% | 79.34% | 90.91% | Gardina^1^(post-hoc) |
| GO terms, D&C | 379 | 169,(44.59%) | 81.64% | **97.52%** | **100.00%** | Soreq on Gardina^1^  (ad-hoc, P<0.05) |
|  | 624 | 206,(33.01%) | **99.52%** | 100.00% | 100.00% | Soreq on Gardina^1^ (P<0.05) + Gardina^1^ combined |

**Table 1. The number of detected BP colon cancer expression data GO categories in each method, and intersections with the other methods.** The number and percent of categories detected by each method compared to the other one is given. The diagonal represents the number of categories detected solely by each method. The results are ordered by detection overlap rate, from the lowest to the highest.

GO analysis results on the different data sets will be given upon request.

References

1. Gardina, P.J. et al. Alternative splicing and differential gene expression in colon cancer detected by a whole genome exon array. *BMC Genomics* **7**, 325 (2006).

2. Maglietta, R. et al. Statistical assessment of functional categories of genes deregulated in pathological conditions by using microarray data. *Bioinformatics* (2007).

3. Bush, C.R. et al. Functional genomic analysis reveals crosstalk between peroxisome proliferator-activated receptor gamma (PPARgamma ) and calcium signaling in human colorectal cancer cells. *J Biol Chem* (2007).

4. Patel, B.B. et al. Searchable high-resolution 2D gel proteome of the human colon crypt. *J Proteome Res* **6**, 2232-2238 (2007).

5. Hosack, D.A., Dennis, G., Jr., Sherman, B.T., Lane, H.C. & Lempicki, R.A. Identifying biological themes within lists of genes with EASE. *Genome Biol* **4**, R70 (2003).
