# Supplementary material for: Identifying Alternative Hyper-Splicing Signatures in MG-Thymoma by Exon Arrays
Source: PLoS One. 2008 Jun 11;3(6):e2392. doi: 10.1371/journal.pone.0002392 (PMC2409220; doi:10.1371/journal.pone.0002392)
Supplement: Text S2 — Global Exons Probe Sets Changes in Different Gene Portions of MG-Thymoma and Colon Cancer. Statistical results of the comparisons between different gene edges (3′ and 5′ regions) expression changes between MG-thymoma to healthy thymuses, and differs from permutated MG-thymoma data set. (0.03 MB DOC) [file pone.0002392.s009.doc]

# Text S2

K = (N1*N2)/(N1 + N2)

**3’ 600 bps compared to 3’ 60 bps:**

600 nts start mean: -0.061451 start 60 mean: -0.10539

600 nts median: -0.019091 start 60 median: -0.0072986

600 nts start N: 16318 2.5% start 60: 2131

KS (N=1885). Smaller P: 0.00020131 Larger P: 0.076566

Non parametric (KW): 0.64999

600 nts Start variance: 1.086 start 60 variance: 1.4683 Variance test P: 0

low KS: 0.00020131 | high KS: 0.076566 | KW: 0.64999 | VAR: 0

**3’ 10% compared to 5’ 10%:**

10% start mean: -0.051413 10% end mean: NaN

10% start median: -0.077654 10% end median: NaN

10% start N: 41806 10% end N: 44540

KS (K =21565). Smaller P: 0.93157 Larger P: 0.00092407

Non parametric (KW): 3.3456e-075

start variance: 1.0386 end variance: NaN Variance test P: 1.0001

**3’ 5% compared to 5’ 5%:**

5% start mean: -0.025107 5% end mean: -0.046926

5% start median: -0.04606 5% end median: -0.060754

5% start N: 26912 5% end N: 26899

KS (K =13453). Smaller P: 0.029991 Larger P: 0.94749

Non parametric (KW): 0.024776

start variance: 0.98878 end variance: 1.0801 Variance test P: 4.2566e-013

**3’ 2.5% compared to 5’ 2.5%:**

2.5% start mean: -0.039185 2.5% end mean: -0.023639

2.5% start median: -0.093109 2.5% end median: -0.031364

2.5% start N: 17195 2.5% end N: 17547

KS (K =8685). Smaller P: 0 Larger P: 0

Non parametric (KW): 0.0061616

start variance: 8.9083 end variance: 1.0155 Variance test P: 0

**80% middle compared to 3’ and 5’ 10%:**

10% start vs. 80% middle:

KS (N =36130). Smaller P: 0.37863 Larger P: 3.9389e-043

Non parametric (KW): 0

80% middle variance: 0.98806 start 10% variance: 1.0386 Variance test P: 1.4521e-011

10% end vs. 80% middle:

KS (N =38153). Smaller P: 0.91279 Larger P: 6.2447e-075

Non parametric (KW): NaN

80% middle: 0.98806 end 10% variance: NaN Variance test P: 0.99863

**Permutations on log ratios:**

MG-Thymoma change VS. Perm1:

KS (N =9222). Smaller P: 0.99213 Larger P: 0

Non parametric (KW): NaN

start variance: NaN end variance: 0.16891 Variance test P: 1.0005

MG-Thymoma change VS. Perm2:

KS (N =9222). Smaller P: 1 Larger P: 0

Non parametric (KW): NaN

start variance: NaN end variance: 0.14502 Variance test P: 1.0005

PERM 1 low KS: 0.99213 | high KS: 0 | KW: NaN | VAR: 1.0005

PERM 2 low KS: 1 | high KS: 0 | KW: NaN | VAR: 1.0005

----------------------
