# Supplementary material for: Identifying Alternative Hyper-Splicing Signatures in MG-Thymoma by Exon Arrays
Source: PLoS One. 2008 Jun 11;3(6):e2392. doi: 10.1371/journal.pone.0002392 (PMC2409220; doi:10.1371/journal.pone.0002392)
Supplement: Text S4 — The Intron in which the New ACHE Exon is Located is Enriched with ALu Repeats. Results of repeat masker [53] on the constitutive ACHE exons number 2 and 3, intron 2–3 and intron 3–4 (where the new exon is located). (0.04 MB DOC) [file pone.0002392.s010.doc]

# Text S4

ACHE_Exon_2:

RepeatMasker ResultsRepeatMasker started 02-May-2007 06:13:47 PDT

RepeatMasker version open-3.1.6

Search engine: Crossmatch

analyzing file /usr/local/rmserver/tmp/RM2sequpload_1178111617

Checking for E. coli insertion elements

identifying simple repeats in batch 1 of 1

identifying full-length ALUs in batch 1 of 1

identifying full-length interspersed repeats in batch 1 of 1

identifying remaining ALUs in batch 1 of 1

identifying most interspersed repeats in batch 1 of 1

identifying long interspersed repeats in batch 1 of 1

identifying ancient repeats in batch 1 of 1

identifying retrovirus-like sequences in batch 1 of 1

identifying tough LINE1s in batch 1 of 1

identifying more simple repeats in batch 1 of 1

identifying low complexity regions in batch 1 of 1

No repetitive sequences were detected in /usr/local/rmserver/tmp/RM2sequpload_1178111617

11.89user 2.12system 0:14.24elapsed 98%CPU (0avgtext+0avgdata 0maxresident)k

0inputs+0outputs (9714major+134628minor)pagefaults 0swaps

Results

Right-click and select "Save As" to save results to your computer or click on

the link to view the file in the browser.

Annotation File:RM2sequpload_1178111617.out

ACHE_Exon_3:

RepeatMasker ResultsRepeatMasker started 02-May-2007 06:17:50 PDT

RepeatMasker version open-3.1.6

Search engine: Crossmatch

analyzing file /usr/local/rmserver/tmp/RM2sequpload_1178111860

Checking for E. coli insertion elements

identifying simple repeats in batch 1 of 1

identifying full-length ALUs in batch 1 of 1

identifying full-length interspersed repeats in batch 1 of 1

identifying remaining ALUs in batch 1 of 1

identifying most interspersed repeats in batch 1 of 1

identifying long interspersed repeats in batch 1 of 1

identifying ancient repeats in batch 1 of 1

identifying retrovirus-like sequences in batch 1 of 1

identifying tough LINE1s in batch 1 of 1

identifying more simple repeats in batch 1 of 1

identifying low complexity regions in batch 1 of 1

No repetitive sequences were detected in /usr/local/rmserver/tmp/RM2sequpload_1178111860

10.13user 2.00system 0:12.33elapsed 98%CPU (0avgtext+0avgdata 0maxresident)k

0inputs+0outputs (9714major+134488minor)pagefaults 0swaps

Results

Right-click and select "Save As" to save results to your computer or click on

the link to view the file in the browser.

Annotation File:RM2sequpload_1178111860.out

ACHE intron 2-3:

RepeatMasker ResultsRepeatMasker started 02-May-2007 06:11:52 PDT

RepeatMasker version open-3.1.6

Search engine: Crossmatch

analyzing file /usr/local/rmserver/tmp/RM2sequpload_1178111501

Checking for E. coli insertion elements

identifying simple repeats in batch 1 of 1

identifying full-length ALUs in batch 1 of 1

identifying full-length interspersed repeats in batch 1 of 1

identifying remaining ALUs in batch 1 of 1

identifying most interspersed repeats in batch 1 of 1

identifying long interspersed repeats in batch 1 of 1

identifying ancient repeats in batch 1 of 1

identifying retrovirus-like sequences in batch 1 of 1

identifying tough LINE1s in batch 1 of 1

identifying more simple repeats in batch 1 of 1

identifying low complexity regions in batch 1 of 1

processing output:

cycle 0A

cycle 1

cycle 2

cycle 3

cycle 4

masking

done

24.97user 1.99system 0:27.53elapsed 97%CPU (0avgtext+0avgdata 0maxresident)k

0inputs+0outputs (10156major+135725minor)pagefaults 0swaps

Summary:

==================================================

file name: RM2sequpload_1178111501

sequences: 1

total length: 3697 bp (3697 bp excl N/X-runs)

GC level: 43.31 %

bases masked: 276 bp ( 7.47 %)

==================================================

number of length percentage

elements* occupied of sequence

--------------------------------------------------

SINEs: 1 276 bp 7.47 %

ALUs 1 276 bp 7.47 %

MIRs 0 0 bp 0.00 %

LINEs: 0 0 bp 0.00 %

LINE1 0 0 bp 0.00 %

LINE2 0 0 bp 0.00 %

L3/CR1 0 0 bp 0.00 %

LTR elements: 0 0 bp 0.00 %

MaLRs 0 0 bp 0.00 %

ERVL 0 0 bp 0.00 %

ERV_classI 0 0 bp 0.00 %

ERV_classII 0 0 bp 0.00 %

DNA elements: 0 0 bp 0.00 %

MER1_type 0 0 bp 0.00 %

MER2_type 0 0 bp 0.00 %

Unclassified: 0 0 bp 0.00 %

Total interspersed repeats: 276 bp 7.47 %

Small RNA: 0 0 bp 0.00 %

Satellites: 0 0 bp 0.00 %

Simple repeats: 0 0 bp 0.00 %

Low complexity: 0 0 bp 0.00 %

==================================================

* most repeats fragmented by insertions or deletions

have been counted as one element

The query species was assumed to be Homo

RepeatMasker version open-3.1.6 , default mode

run with cross_match version 0.990329

RepBase Update 20061006, RM database version 20061006

Results

Right-click and select "Save As" to save results to your computer or click on

the link to view the file in the browser.

Annotation File:RM2sequpload_1178111501.out

Masked File:RM2sequpload_1178111501.masked

ACHE intron 3-4:

RepeatMasker ResultsRepeatMasker started 02-May-2007 06:07:39 PDT

RepeatMasker version open-3.1.6

Search engine: Crossmatch

analyzing file /usr/local/rmserver/tmp/RM2sequpload_1178111249

Checking for E. coli insertion elements

identifying simple repeats in batch 1 of 1

identifying full-length ALUs in batch 1 of 1

identifying full-length interspersed repeats in batch 1 of 1

identifying remaining ALUs in batch 1 of 1

identifying most interspersed repeats in batch 1 of 1

identifying long interspersed repeats in batch 1 of 1

identifying ancient repeats in batch 1 of 1

identifying retrovirus-like sequences in batch 1 of 1

identifying tough LINE1s in batch 1 of 1

identifying more simple repeats in batch 1 of 1

identifying low complexity regions in batch 1 of 1

processing output:

cycle 0A

cycle 1

cycle 2

cycle 3

cycle 4

masking

done

10.72user 1.84system 0:13.05elapsed 96%CPU (0avgtext+0avgdata 0maxresident)k

0inputs+0outputs (10156major+135510minor)pagefaults 0swaps

Summary:

==================================================

file name: RM2sequpload_1178111249

sequences: 1

total length: 995 bp (995 bp excl N/X-runs)

GC level: 52.66 %

bases masked: 304 bp ( 30.55 %)

==================================================

number of length percentage

elements* occupied of sequence

--------------------------------------------------

SINEs: 1 304 bp 30.55 %

ALUs 1 304 bp 30.55 %

MIRs 0 0 bp 0.00 %

LINEs: 0 0 bp 0.00 %

LINE1 0 0 bp 0.00 %

LINE2 0 0 bp 0.00 %

L3/CR1 0 0 bp 0.00 %

LTR elements: 0 0 bp 0.00 %

MaLRs 0 0 bp 0.00 %

ERVL 0 0 bp 0.00 %

ERV_classI 0 0 bp 0.00 %

ERV_classII 0 0 bp 0.00 %

DNA elements: 0 0 bp 0.00 %

MER1_type 0 0 bp 0.00 %

MER2_type 0 0 bp 0.00 %

Unclassified: 0 0 bp 0.00 %

Total interspersed repeats: 304 bp 30.55 %

Small RNA: 0 0 bp 0.00 %

Satellites: 0 0 bp 0.00 %

Simple repeats: 0 0 bp 0.00 %

Low complexity: 0 0 bp 0.00 %

==================================================

* most repeats fragmented by insertions or deletions

have been counted as one element

The query species was assumed to be Homo

RepeatMasker version open-3.1.6 , default mode

run with cross_match version 0.990329

RepBase Update 20061006, RM database version 20061006

Results

Right-click and select "Save As" to save results to your computer or click on

the link to view the file in the browser.

Annotation File:RM2sequpload_1178111249.out

Masked File:RM2sequpload_1178111249.masked
