# Supplementary material for: Identifying Alternative Hyper-Splicing Signatures in MG-Thymoma by Exon Arrays
Source: PLoS One. 2008 Jun 11;3(6):e2392. doi: 10.1371/journal.pone.0002392 (PMC2409220; doi:10.1371/journal.pone.0002392)
Supplement: Text S3 — Core Probe Sets Normalized Signals Intensity of Detected MG-Thymoma genes. Expression signals intensity of core probe sets of the MG-thymoma genes detected by the specific term to parent ad-hoc approach combined with fold change threshold are given for MG-thymoma and healthy thymuses. (0.09 MB DOC) [file pone.0002392.s011.doc]

# Text S3

Y axis are normalized and summarized signal intensities

X axis are the core probe sets

T – MG-thymoma

N-Normal thymus
