# Supplementary material for: Identifying Alternative Hyper-Splicing Signatures in MG-Thymoma by Exon Arrays
Source: PLoS One. 2008 Jun 11;3(6):e2392. doi: 10.1371/journal.pone.0002392 (PMC2409220; doi:10.1371/journal.pone.0002392)
Supplement: Text S5 — Additional materials and methods (0.04 MB DOC) [file pone.0002392.s012.doc]

**Text S5**

**Additional materials and methods**

**Protein isolation**

Using Sigma T9424 TRI REAGENT (Sigma, Saint Louis, Missouri, USA) – a mixture of guanidine thiocyanate and phenol in mono-phase solution, the thymus tissues were homogenized in TRI REAGENT (1 ml per 50-100 mg of tissue) in a Polytron homogenizer and light vertex and incubation (10 min, room temp). 0.2 ml of chloroform was added per ml of TRI REAGENT and incubation (10 min, room temp). Resulting mixture centrifuged at 12,000 x g for 15 min at 4 °C. Centrifugation separated the mixture into 3 phases: protein, DNA and RNA. The upper RNA level was removed. 300 μl ethanol per 1μl TRI REAGENT was added. The upper, protein layer, moved to 15ml ependorf tube and precipitate from the phenol-ethanol supernatants using 1.5ml isopropanol per 1ml reagent. Light vertex, and incubation (10 min., room temp.), and then centrifuged (10,254g, 10 min at 4 °C).

**In-gel proteolysis**

Proteins subjected to gel electrophoresis were reduced with 10 mM DTT (60ºC, 30 min) and modified with 100 mM iodoacetamide in 10 mM ammonium bicarbonate (room temp, 30 min). Gel pieces were dehydrated with acetonitrile, rehydrated with 10% acetonitrile in 10 mM ammonium bicarbonate containing modified trypsin ((Promega, at a 1:100 enzyme-to-substrate ratio), and incubated (overnight, 37^o^C). Resultant peptides were recovered and analyzed.

**Mass spectrometry (MS) analysis**

Tryptic peptides prepared from gel-separated spots were resolved by reverse-phase chromatography on 0.1 X 200-mm fused silica capillaries (J&W, 100 micrometer ID) packed with Everest reversed phase material (Grace, Vydac, CA). The peptides were eluted with linear 50 minutes gradients of 5 to 95% acetonitrile with 0.1% formic acid in water at flow rates of 0.4 μl/min. MS was performed by an ion-trap mass spectrometer (LCQ-DecaXP, Finnigan, San Jose, CA) in a positive mode using repetitively full MS scan followed by collision-induced dissociation (CID) of the 3 most dominant ions selected from the first MS scan. The MS data was clustered and analyzed using the Sequest software (J. Eng and J. Yates, University of Washington and Finnigan, San Jose) or Pep-Miner[1] searching the NR-NCBI database. Results on 1D gels are given under ST6 and on 2D gels under ST7. K-means clustering performed on 1D MS gel results, data is given under AF8.

Mass spectrometry (MS) data analysis

MS analysis identified approximately 1800 protein isoforms using the NCBI database of 5 species[1], the large majority identified in human in addition to other species. A total of 241 signal values was accepted for all the predicted proteins. These values were analyzed using their change level in the single dimension gel (1D) analysis (Supplementary Table 4). K-Means clustering was implemented using Matlab, and was run with 100 iterations on the identified human proteins. For proteins of which more then one isoform was identified, one isoform was selected to represent the protein name in the analysis. Numerical data consisted of the number of identified peptides for each of the proteins. The mean number of identified peptides was calculated for MG-thymoma and healthy samples separately. The algorithm divided each group (MG-thymoma/ healthy) to 4 distinct clusters by finding 4 centers (Supplementary Material). Proteins that belonged to different groups in patients and control samples were excluded, which reduced the number of changed proteins to 91. We then crossed the identified proteins with the data obtained from 2D gel MS analysis (AF9 and AF10) and found [2] genes which were present in both. The 1D identified NCBI proteins (Gene Identifiers - GIs). Downstream K-means clustering served to identify MG thymoma-associated modulations. As the Affymetrix annotation files directly link genes by names to these terms, we used these files to construct a MySQL database. We then mapped the detected proteins to their GO terms, entered into the database, and connected to the corresponding GO terms. For this purpose, MySQL queries were entered into the exon array annotation data in the database (originated from transcript_annot Affymetrix file) by mapping each protein to its corresponding exon array transcript and GO terms by SQL queries. ENSEMBL database (H.Sapians March 2006) was downloaded from ENSEMBL genome browser to MySQL database and *ACHE* exons and introns sequences obtained from it.

*References*

1. Beer I, Barnea E, Ziv T, Admon A (2004) Improving large-scale proteomics by clustering of mass spectrometry data. Proteomics 4: 950-960.

2. Cheng J, Kapranov P, Drenkow J, Dike S, Brubaker S, et al. (2005) Transcriptional maps of 10 human chromosomes at 5-nucleotide resolution. Science 308: 1149-1154.
